# A Smart Headband for Multimodal Physiological Monitoring in Human Exercises

**DOI:** 10.1002/advs.74279

**Published:** 2026-02-08

**Authors:** Shiqiang Liu, Guanxing Chen, Jingyi Peng, Shuo Tian, Qian Mao, Caise Wei, Jinfeng Yuan, Rong Zhu

**Affiliations:** ^1^ State Key Laboratory of Precision Measurement Technology and Instruments Department of Precision Instrument Tsinghua University Beijing China

## Abstract

Wearable multimodal physiological monitoring plays a crucial role in sports training and contest, personalized health management, and exercise rehabilitation. However, challenges in continuous and accurate physiological signal acquisition, motion artifact in human exercises, and wearable convenience with multimodal integration, hinder its practical applications. Here, we propose a smart headband using a thermal‐sensation‐based electronic skin (e‐skin) for continuously and accurately monitoring multimodal physiological parameters, including pulse pressure waveform, total metabolic energy cost, heart rate, and forehead temperature. The headband allows accurate and continuous monitoring under both static and dynamic conditions for indoor and outdoor activities in real life. The effectiveness and robustness of multimodal physiological monitoring is validated across various human activities including resting, walking, jogging, bicycling, hill climbing and even long‐distance running. Significant physiological variations with fatigue are uncovered, potentially revealing rich information about cardiac load, cardiovascular condition, and potential risk.

## Graphical abstract

A novel smart headband incorporating a thermal‐sensation‐based electronic skin is presented for continuous and accurate multimodal physiological monitoring, including pulse waveforms, total metabolic energy expenditure, heart rate, and forehead temperature, across both static and dynamic daily activities.

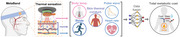

## 1 Introduction

Personalized health monitoring and management has been highly emphasized in nowadays [1, 2, 3]. Insufficient exercise brings lots of health problems, such as obesity [4], stroke [5], and some chronic diseases like type II diabetes [6]. On the other hand, overexercising also causes damages on heart [7], muscles [8], endocrine and immune systems [9, 10], and even leads to sudden death. For example, Stephen Shanks—A British runner with extensive marathon experience, passed away suddenly after completing the London Marathon. Human health risks highlight the importance of personalized real‐time assessment and scientific management of health, emphasizing the urgent need for seamless tools that enable continuous daily monitoring of essential physiological information in both static and dynamic scenarios.

Total metabolic rate (including resting and active metabolic rates), pulse pressure waveform and forehead temperature are key physiological parameters in health assessment and management [11, 12]. Metabolic rate from physical activity reveals both exercise intensity and energy expenditure [13, 14]. Resting metabolic rate helps quantify an individual's baseline caloric requirements, and serves an indicator for diagnosing metabolic disorders such as hyperthyroidism or hypothyroidism [15]. Continuous monitoring of the total metabolic rate and energy cost during daily activities including both static and active situations is necessary for quantitative evaluation and management of personal health. Pulse pressure waveform implies a lot of cardio‐cerebrovascular conditions [16], such as hypertension and arteriosclerosis. Forehead temperature can indicate infection [17]. Besides, pulse waveform and forehead temperature reflect real‐time exercise intensity, and unveils insights into possible risks, such as heatstroke from overheating [18, 19, 20], and abnormal heartbeat from overfatigue [21], during exercise or physical labor. By comprehensively analyzing the multimodal physiological information, a more holistic understanding of an individual's health status can be achieved. In addition, real‐time monitoring is crucial for providing timely warnings and enabling prompt responses to sudden health risks.

Advances in materials, fabrication, sensor design, and machine learning have led to significant breakthroughs in wearable electronics for physical and physiological monitoring [22, 23, 24, 25]. However, three major challenges remain for achieving continuous, real‐time and simultaneous monitoring of these physiological parameters. The first is the lack of wearable tools for accurate acquisition of the key information. For example, existing methodologies are insufficient for developing wearable devices capable of real‐time monitoring of both resting and active metabolic rates [26, 27]. Although the methods based on accelerometers, IMUs [28, 29, 30, 31, 32, 33], heart rate and heat flux sensors [27] have been investigated, they usually work under some specific scenarios (e.g., normal locomotion [28]), and are quite insufficient and inaccurate under real‐life complex condition [33]. At present, quantitative estimation of both resting and active metabolic rate still relies on traditional bulky and expensive instruments, such as the indirect calorimetry method [34, 35]. The second challenge for these wearable systems is ensuring they function reliably and continuously in complex real‐life settings, especially during high‐intensity activities. For example, pulse waveform monitoring is seriously affected by motion artifacts during dynamic situations. This is a common issue in physiological monitoring and has significantly hindered its practical application [36, 37]. The third challenge is the integration of incompatible multimodal physiological monitoring methods in a compact wearable system. At present, wearable metabolic rate estimation depends on sensors like accelerometers, IMUs, heart rate sensors and heat flux sensors. Wearable pulse waveform monitoring usually uses photoplethysmography (PPG) [38] sensors, pressure sensors [16, 39, 40], strain sensors [41, 42], piezoelectric sensors [43], triboelectric sensors [44] and so on. And temperature measurement relies on metal or semiconductor thermometers. These sensors are incompatible with each other for their fabrication process, signal conditioning circuits and monitoring locations (for example, positions of interest are usually radial/carotid artery for pulse waveform, but are usually oral/axillary/temporal skin for body temperature). This hinders the design of compact, low‐cost wearable systems capable of continuously monitoring multimodal physiological information. Therefore, it's still a big challenge to monitor these multimodal physiological parameters simultaneously and continuously in real time using wearable interfaces with easy setup.

**FIGURE 1:**
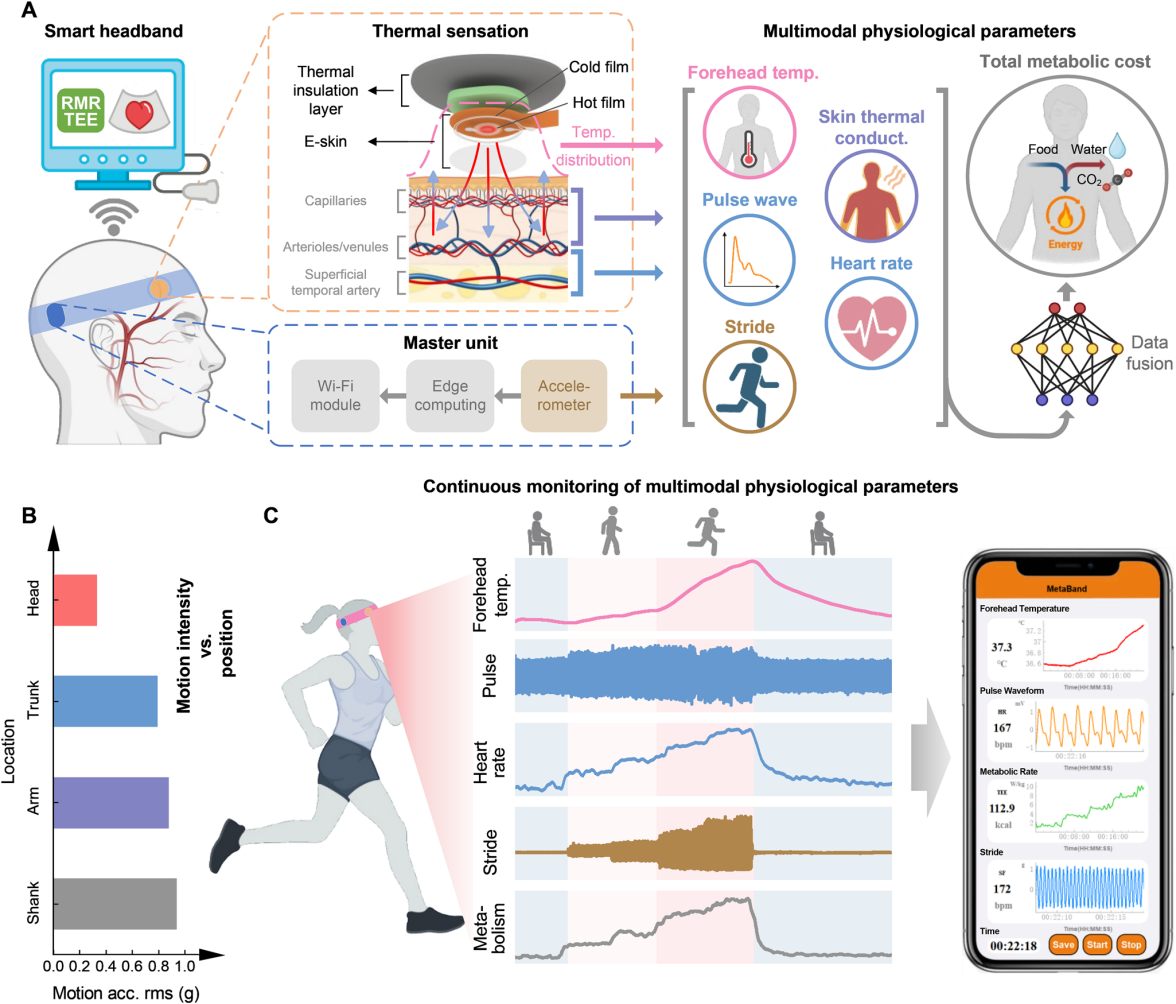
A smart headband for real‐time and in‐situ multimodal physiological monitoring. (A) The smart headband is able to monitor physiological information by the e‐skin, including skin thermal conductivity, pulse waveform, heart rate and the forehead temperature, and allows real‐time estimation of total metabolic energy cost. The system design of the proposed smart headband, including the textile headband, the multimodal sensing e‐skin, and the master unit with a built‐in accelerometer and an edge computing module. (B) Head has lower motion interference intensity than other typical locations on human body (naturally running). (C) Multimodal physiological parameters can be estimated and displayed on smart phones in real time under both resting and active scenarios.

In this paper, we propose a smart headband (named MetaBand) using a thermal‐sensation‐based electronic skin (e‐skin) for continuously and accurately monitoring multimodal physiological information, including pulse waveform, total metabolic energy cost, heart rate, and forehead temperature, during both static and dynamic daily activities. The e‐skin allows multimodal physiological sensation using a very compact design of sensing structure (two Pt thin‐film thermistors on a flexible polyimide substrate, one is electrically heated and called hot film, the other is cold film and acts as a temperature sensor, as shown in Figure 1A). The hot film detects the forehead skin heat conductivity and its variation induced by arterial pulse. The cold film detects the forehead skin temperature that is related to body temperature. Apart from the headband's inherent self‐stabilization (Figure 1B), we propose an adaptive filtering method onto the hot film signals to eliminate motion artifacts and extract the high‐fidelity pulse pressure waveforms and accurate heart rates during high‐intensity exercise. Leveraging the strong circulatory system of human head and the environmental thermal insulation of the headband, the forehead skin temperature measured by cold film is validated to be an effective indicator of human body temperature. The accurate pulse waveform and forehead temperature provides key clues of the intensity of human physiological activities. By combining the multimodal physiological information (skin thermal conductivity, pulse waveform, forehead temperature, etc.) from the e‐skin and human body activity information from a built‐in accelerometer, the total metabolic rate of human body, including resting and active metabolic rate, can be continuously and accurately estimated in real time (Figure 1A). We validate the effectiveness and robustness of the wearable system in various human activities including resting, walking, jogging, bicycling, hill climbing and even long‐distance running. Compared to existing wearable systems, our method exhibits significant advantages in multimodality and accuracy utilizing a compact headband compatible for daily use. The headband allows continuous and accurate monitoring of significant physiological parameters simultaneously under both static and dynamic conditions for indoor and outdoor activities in real life (Figure 1C). In particular, experimental results showing how pulse pressure waveform features varying with fatigue (e.g., the pulse waveform amplitude, the reflective peak, and the related timings) reveal rich information about cardiac load, cardiovascular condition, and potential risk. The MetaBand provides a promising tool for not only daily health management, but also continuous and comprehensive investigation on cardiovascular disease pathogenesis.

## 2 Design of the Smart Headband

**FIGURE 2:**
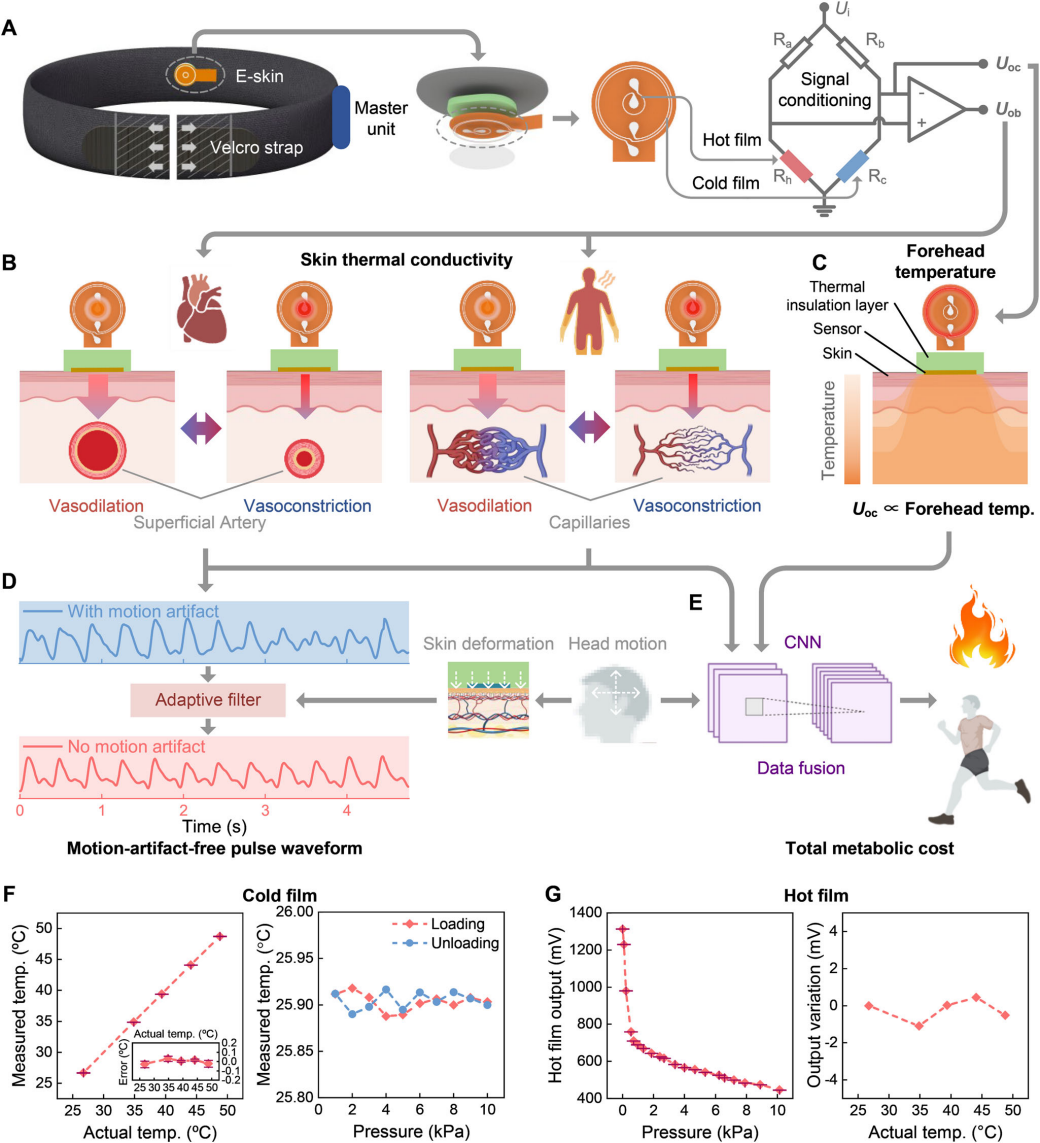
Multimodal sensation of the e‐skin based on thermal sensation principle. (A) Details of the e‐skin, including the structure and conditioning circuit based on Wheatstone bridge. (B) The working principle for monitoring pulse waveform due to superficial temporal artery vasodilation/vasoconstriction, and heat‐dissipation‐related skin blood perfusion due to capillaries vasodilation/vasoconstriction. (C) The working principle for forehead temperature estimation using the skin temperature, due to the strong circulatory system of human head skin and the environmental heat flux blocking configuration of the e‐skin. (D) Principle of motion‐artifact‐free pulse waveform extraction. (E) Principle of real‐time total metabolic energy consumption estimation. (F) The performance of the cold film for temperature measurement. And the impact of pressure on temperature measurement. (G) The pressure response of the hot film on human skin. And the impact of environment temperature on the hot film output.

The multimodal physiological monitoring system adopts a headband design (named MetaBand) for long‐term continuous daily wear, as shown in Figure 2A and Figure S1. The MetaBand consists of an elastic textile band, an e‐skin and a master unit. The band tension is adjustable for different users by using the Velcro strap. The e‐skin is fixed on the inner side of the band. During monitoring, the e‐skin is conformally attached on the skin over superficial temporal artery for multimodal physiological monitoring. The master unit contains a built‐in accelerometer, an edge computing module, and a Wi‐Fi module. The built‐in accelerometer is used to monitor human body activity. Head monitoring devices offer advantages over those placed on the trunk and limbs. First, the temporal has thin skin, low fat, and a rich network of blood vessels, making them ideal for heat detection. For this reason, the forehead skin temperature has been widely used as an indicator of body temperature [45]. Second, the sensors placed on the head experience fewer motion artifacts compared to those on the body and limbs, thanks to the head's natural stabilization mechanisms, as well as the vibration damping provided by the soft tissues and bone structures of human body, as shown in Figure 1B. Compared to conventional wearable solutions on body or limbs, our headband design allows in‐situ monitoring of multimodal physiological information, including pulse pressure waveform, skin thermal conductivity, forehead temperature, and head movement, enabling to accurately estimate total energy cost level by combining the multimodalities. We allow more comprehensive understanding of human body status.

## 3 Working Principle of Multimodal Sensation

We develop a flexible e‐skin for real‐time monitoring of multimodal physiological signals, including the skin thermal conductivity and forehead temperature, pulse waveform, and the total energy consumption of human body. The e‐skin has a very compact design of sensing structure including two Pt thin‐film thermistors on a flexible polyimide substrate (Figure 2A and Figure S2), one of which is electrically heated and called hot film (70 Ω, about 5 °C higher than human skin), the other is cold film (1.2 kΩ) with negligible electrical heating and acts as a temperature sensor. The hot film serves as both a Joule heater and its own temperature detector, which is sensitive to its surrounding (i.e., skin) heat conduction variation. As shown in Figure 2A, the e‐skin is driven by a Wheatstone bridge. *U*_ob_ and *U*_oc_ are used as the outputs of the hot film and the cold film respectively. Benefiting from the monolithic integration of the cold film and hot film, the environmental temperature impact on the hot film is eliminated. In other words, the hot film's output is immune from the surrounding temperature variation.

The hot film is affixed to the forehead skin and its heat loss is regulated by the skin's thermal conductance (Figure 2B). Specifically, the hot film's heat loss is enhanced when the skin thermal conductivity is higher, and reduced when the skin thermal conductivity is lower. Human skin has a layered structure, including epidermis, dermis and hypodermis, with different tissues and vascular networks in each layer. The pressure changes the relative distribution and volume fraction of components in the skin, leading to a corresponding heat conductivity variation of the skin. We call this mechanism the piezo‐thermic transduction effect, which refers to the conversion of a pressure stimulus to the heat conductance change of the skin. Specifically, superficial arteries’ vasoconstriction and diastole due to the heart pulsation compresses and relaxes the superficial skin tissue above them, and will cause a periodic variation on the skin's heat conductivity. Additionally, the blood perfusion variation caused by capillaries vasodilation/vasoconstriction also alters the skin's heat conductivity, which always has a direct correlation with the level of human skin's heat dissipation and body's energy consumption.

The cold film measures the forehead skin's temperature. The e‐skin is covered by a PDMS layer and the textile headband to suppress the heat exchange between the sensors and the external environment. Leveraging the strong circulatory system of human head and the environmental thermal insulation of the headband, the forehead skin temperature measured by cold film can be used as an indicator of human body temperature (Figure 2C). The effect of environmental heat flux insulation of the headband is validated by using a simulation (COMSOL Multiphysics 6.1) in Figure S3A. The simulation results show that the subcutaneous temperature keeps 37.0 °C. When the ambient temperature changes from 10 °C to 35 °C, the average temperature difference between the e‐skin (the bottom side of the polyimide) and the subcutaneous temperature keeps less than 0.23 °C (Figure S3B).

The measurements of forehead skin thermal conductivity and forehead temperature offer key insights into the intensity of human physiological activities and the demand for heat dissipation. Therefore, more comprehensive and important physiological information can be derived. First, we extract pulse pressure waveforms from the skin thermal conductivity output. Motion‐artifact‐free pulse waveforms with high fidelity are achieved during even high‐intensity exercise for the first time, by using a proposed adaptive filter (Figure 2D). What's more, the total metabolic energy consumption of human body can be estimated accurately in real time by combining measurements of forehead skin thermal conductivity, forehead temperature, and head motion (Figure 2E). Methods and results of pulse waveform and energy cost monitoring will be discussed in detail later.

The characteristics of the cold film and hot film are shown in Figure 2F, Figure 2G and Figure S4. The cold film's temperature measurement is calibrated on a thermal plate. The cold film output is proportional to the measured temperature, and the error between the actual and measured temperatures is below 0.1°C (Figure 2F). The cold film is also tested for the anti‐interference from pressure stimuli. The cold film's temperature measurement keeps nearly constant while applying and lifting the pressure stimuli (Figure 2F), indicating that the forehead temperature measurement is free from the pulse or motion interference. The pressure‐sensitive response of the hot film (*U*_ob_ in Figure 2A) is tested by applying a pressure onto human skin, and demonstrates a high sensitivity (Figure 2G, using a force gauge, SH‐50, Sundoo Co. Ltd., Wenzhou, China). The hot film has a dynamic response time 60 ms (Figure S5), enabling the high‐fidelity capture of pulse pressure waveforms typically ranging from 0.5 Hz to 5 Hz [46]. The environmental temperature effect on the hot film is also tested (Figure 2G), whose results indicate that the hot film is immune from the environment temperature and works reliably for physiological monitoring.

## 4 Motion Artifact Removal for Continuous Physiological Monitoring

**FIGURE 3:**
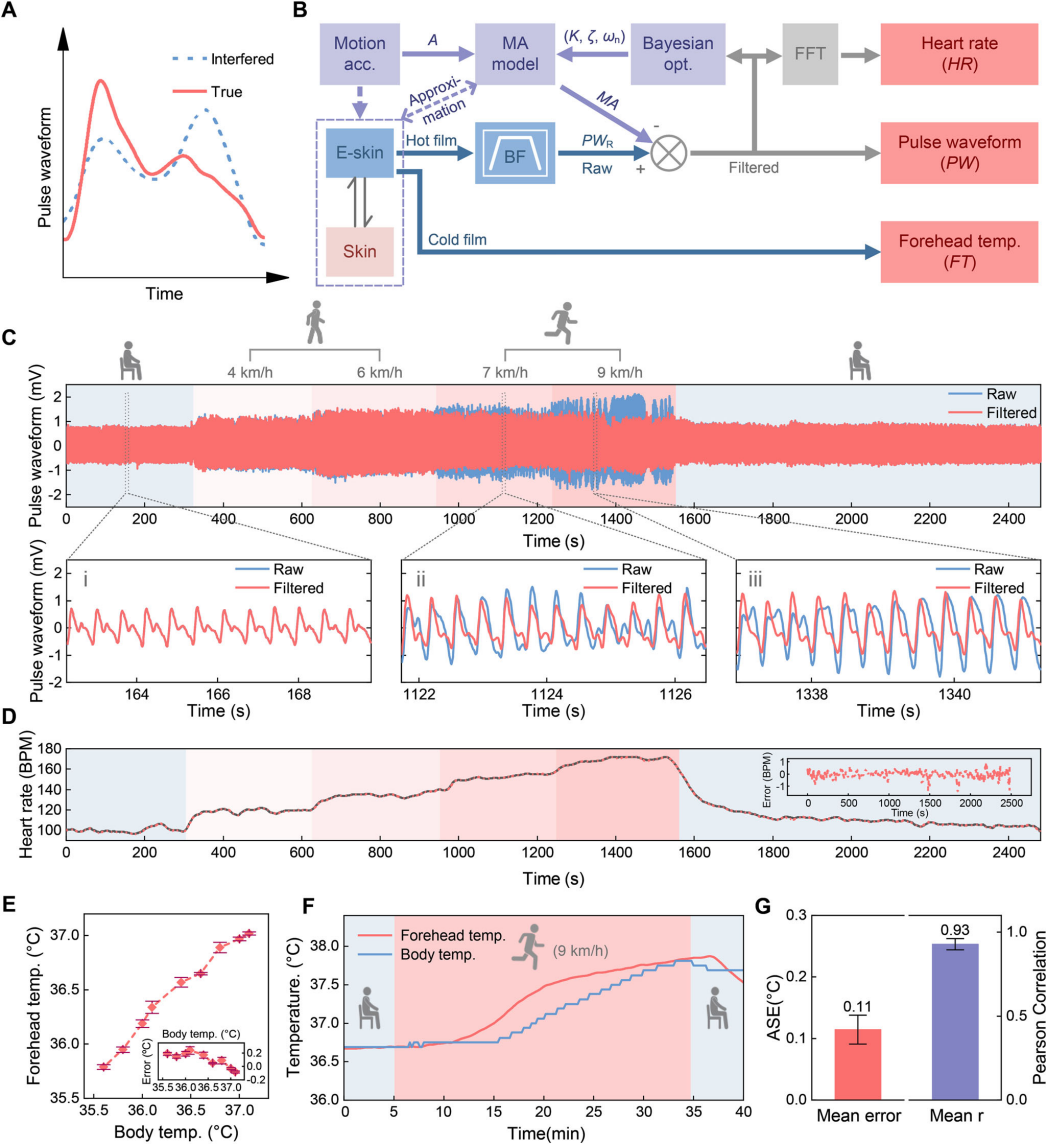
Results of pulse waveform, heart rate and forehead temperature monitoring. (A) True pulse waveform and that interfered by motion artifact. (B) Pulse waveform and heart rate extraction from the hot film output, where BF is band‐pass filter, FFT refers to fast Fourier transform. Motion artifact is removed by our proposed adaptive filtering method based on the biomechanical model of human skin, where *A* is the head movement acceleration, *MA* refers to motion artifact during measured raw pulse waveform *PW*_R_, *K*, ζ and ω_*n*_ are the motion artifact model parameters. The forehead skin temperature is used as an indicator of body temperature here and is directly measured by the cold film. (C) The raw and motion‐artifact‐removed (filtered) pulse waveforms during the mimicked exercise routine. Insets show the pulse waveforms in detail while resting and running. (D) Heart rate during the mimicked exercise routine. (E) Forehead temperature measurement results during the static experiments. (F) Forehead temperature measurement results during the exercise experiments. (G) The error and correlation of forehead temperature by our MetaBand compared to the axillary temperature, during exercise.

The most severe challenge in continuous physiological monitoring, especially pulse waveform monitoring, is the serious motion artifact that occurs during dynamic situations. As mentioned above, the on‐head design of MetaBand enables significant decrease in motion artifact and is therefore more suitable for continuous monitoring of physiological parameters, thanks to the head self‐stabilization and the body's natural damping effects (Figure 1C). Nevertheless, motion artifact is hard to totally avoid, and will worsen physiological monitoring especially under high dynamic activities, such as running. Figure 3A gives one of the raw pulse waveforms recorded during running (the blue dashed line). Compared to the expected true pulse waveform (the red solid line), the recorded pulse waveform distorts due to motion induced artifact.

To deal with this problem, we propose an adaptive filtering method, to further attenuate the residual motion artifact and improve the quality of pulse waveform acquisition, as shown in Figure 3B. Here, the dynamical response of human skin's deformation to the applied disturbance force (linearly correlated with the head movement acceleration) is simplified by a two‐order spring‐damping system. The motion‐induced disturbance pressure applied on the sensor is only less than 0.1 kPa during the exercise, meaning that the hot film's output would approximately linearly change with the motion‐induced skin deformation. Therefore, the mapping from head movement acceleration *A*(*s*) to motion artifact *MA*(*s*) in hot film output can be represented by a simplified two‐order model, whose continuous transfer function is formulated in equation (1). Here, *s* is the Laplace operator, ω_*n*_ is the undamped natural frequency of human skin, ζ refers to damping ratio, and *K* is a constant coefficient depending on the individual and the wearing conditions.

(1) $$MA\;\left(s\right)=\frac{K\omega_{n}^{2}}{s^{2}+2\zeta \omega_{n}s+\omega_{n}^{2}}A\left(s\right)$$

The diagram of our adaptive filter for motion artifact removal is shown in Figure 3B. Here, *PW*_R_ is the raw pulse waveforms containing motion artifacts, *A* is the head movement acceleration measured by the built‐in accelerometer in MetaBand. Acting as the reference signal, *A* is used to reconstruct the motion artifact *MA* through the proposed two‐order model in (1). The expected cleansed pulse waveform *PW* is figured out by subtracting motion artifact *MA* from the raw pulse waveform signal *PW*_R_. The reference input *A* here uses the total motion acceleration of three axes by subtracting the gravity from the total acceleration to avoid the wearing orientation impact.

The key problem is how to determine the motion artifact model parameters *K*, ζ and ω_*n*_. Here, we propose a Bayesian optimization‐based method for parameter recognition by optimizing the similarity between continuously captured pulse waveforms, which can be found in Materials and Methods in detail. When the MetaBand is worn on a user, the coefficients *K*, ζ and ω_*n*_ is first recognized adaptively to the user and the wearing condition by Bayesian optimization through a calibration procedure of 2‐minute natural running. Then, the recognized model can be used for motion artifact removal.

The effectiveness and robustness of our motion artifact removal method is validated by experiments that simulate a typical daily exercise routine. The routine includes initial rest, slow walking at 4 km/h, brisk walking at 6 km/h, light jogging at 7 km/h, intense running at 9 km/h, and final rest. The exercise at each speed lasts about 5 minutes. 7 subjects participate the experiments (results are in Figures S13 to S19, the ambient temperature and humidity 26.1 °C, 26% respectively). Here, a standard ECG chest strap (Polar H10, Finland) is adopted for pulse waveform and heart rate measurement evaluation. The measurement results of pulse waveforms from Sub. 1 are illustrated in Figure 3C, where the blue and red curves refer to raw and adaptively filtered pulse waveforms respectively. Details can be seen in the insets in Figure 3C. The relationship between pulse waveforms and the head movement acceleration during exercise can be found in Figure S6. Our adaptive filter works robustly during different activities, including resting, walking and running with different intensities. Therefore, the pulse waveforms can be acquired with high fidelity even under high dynamic motions, by using the MetaBand. Heart rate is further extracted from the filtered pulse waveforms through FFT (fast Fourier transform, Figure 3B), and compared to the standard ECG chest strap, whose results are shown in Figure 3D. The average error of heart rate measurement among participants by using the MetaBand is only 0.44 BPM (*N* = 7) during the whole exercise period (Table S1). Accurate heart rate measurement further quantitatively validates the high quality of pulse waveform acquisition.

Then the continuous measurement capability of forehead temperature is tested during both static and high dynamic exercise situations. One participant takes part in the real‐world static experiments. Here, an ear thermometer (IRT6525, braun, Germany) is used for comparison. He first stays outdoor (10.0 °C, humidity 54%) for about half an hour, and then moves into a warm room (30.0 °C, humidity 32%) and stays for an hour. The cold film on the e‐skin directly measures the forehead skin temperature (Figure 3B), and the in‐ear temperature is tested and recorded simultaneously using the ear thermometer. The testing results are summarized in Figure 3E, with outdoor and indoor phases shown in detail in Figure S7. We can see that, the forehead temperature measured by the MetaBand correlates linearly with the in‐ear body temperature. The deviation is less than 0.24 °C. The forehead temperature is also compared with the in‐ear temperature (IRT6525, braun, Germany) under 4 °C real‐world outdoor conditions (one participant). As shown in Figure S8, the measured forehead temperature varies consistently with the in‐ear temperature with the deviation less than 0.30 °C, while the subject moves from a warm indoor scenario (25.0 °C, humidity 18%) to a cold outdoor scenario (4.0 °C, humidity 39%) with natural airflow. Three participants take part in the exercise experiments (the ambient temperature and humidity 25.1 °C, 26% respectively), including 5‐minute initial rest, 30‐minute intense running at 9 km/h, and 5‐minute final rest. A commercial continuous axillary thermometer (VV‐200, vivalnk, Hangzhou, China) is for reference. Results of one participant are shown in Figure 3F (The other participants are shown in Figures S9 and S10), where the red and blue curves refer to results of our MetaBand and the reference respectively. The forehead temperature measured by MetaBand exhibits similar trend and amplitude to the axillary body temperature. What's more, forehead temperature responds to movement more quickly than axillary temperature, showing advantages in real‐time body temperature monitoring. Results of the participants (*N* = 3) during exercise experiments are summarized in Figure 3G. The MetaBand forehead temperature measurements have a good correlation with the axillary body temperature (Average Pearson correlation coefficient > 0.92), with the ASE (Average Signed Error) less than 0.12 °C (Table S2). Experiments validate that the forehead temperature measured by our MetaBand can be used as an indicator of body temperature.

Therefore, the MetaBand works robustly and reliably in continuously monitoring multimodal physiological parameters, including pulse waveform, heart rate, and forehead temperature, for both static and high dynamic activities.

## 5 Real‐time Estimation of the Total Metabolic Rate

Heat dissipation for body temperature maintenance accounts for almost half of the total energy cost of human body. It is positively correlated with the level of human basic philological (e.g., heartbeat, respiration, nerve activity, etc.) and physical (e.g., muscle contraction, etc.) activities [47], and has been used as the gold standard for the total metabolic energy cost estimation of human body, which is the so‐called direct calorimetry method. The existing instrument for measuring metabolic rate is complex and bulky, which hinders its personal applications.

**FIGURE 4:**
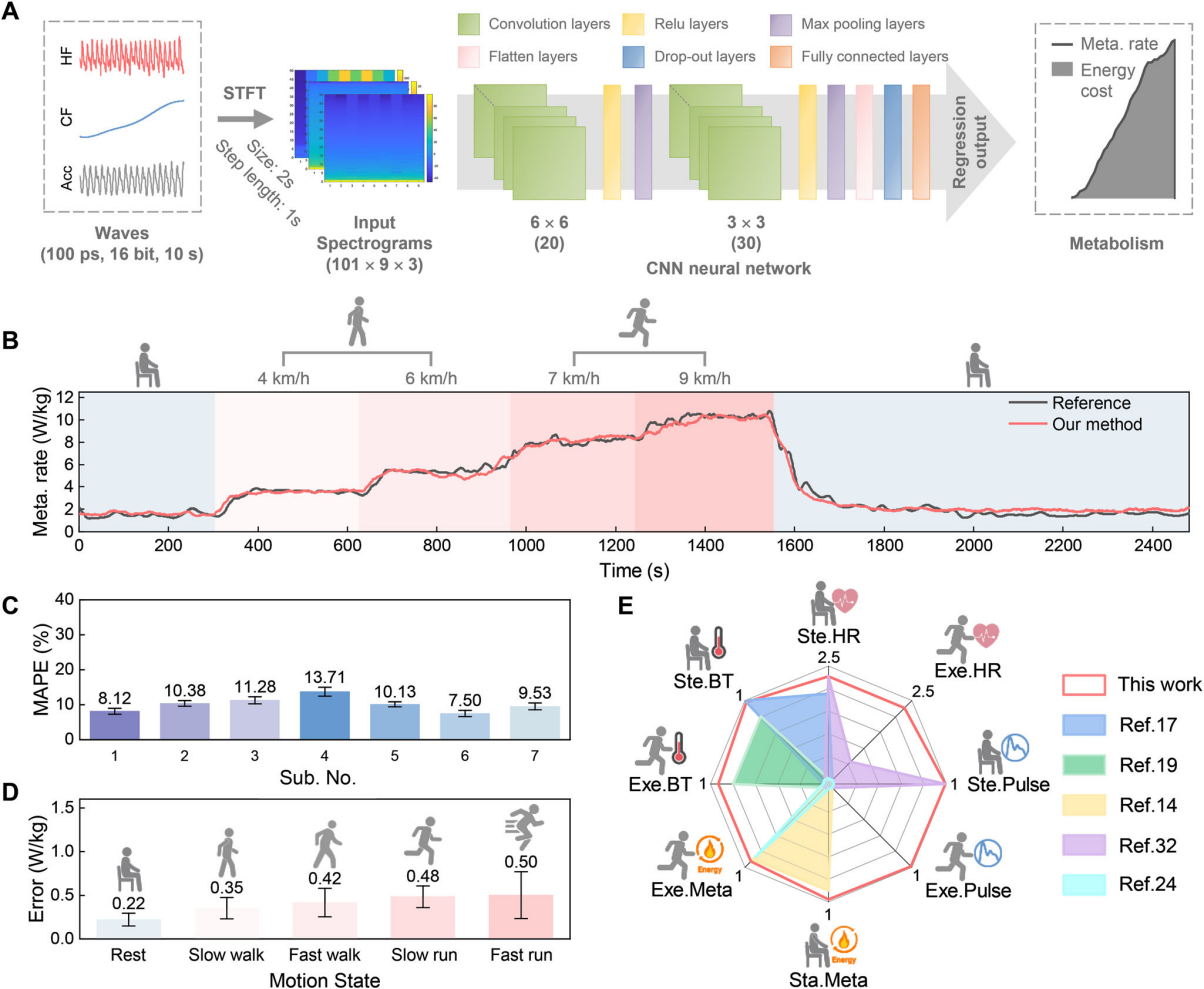
The results of total metabolic energy cost estimation and the comparison between MetaBand and existing wearable methods for multimodal physiological monitoring. (A) The CNN‐based neural network model for real‐time estimation of total metabolic energy cost. HF and CF refer to raw outputs of the hot film and the cold film respectively, Acc refers to the acceleration. (B) Estimation results of total metabolic rate during the mimicked exercise routine including resting, walking and running. (C) The error of total metabolic energy cost estimation among participants. (D) The mean error of total metabolic energy cost during activities with different intensities (*N* = 7). (E) The comparison of multimodal physiological monitoring between our MetaBand and other existing wearable methods, where Ste. HR and Exe. HR refer to heart rate measurements under steady and exercise activities respectively, Ste. Pulse and Exe. Pulse means pulse waveform captures under steady and exercise activities respectively, Sta. Meta and Exe. Meta refers to metabolic rate estimation under static and exercise situations respectively, Ste. BT and Exe. BT refer to body temperature measurements under steady and exercise activities respectively. The metrics of heart rate, pulse waveform, metabolic rate and body temperature used here are the reciprocal of AAE, pulse monitoring capability, the reciprocal of MAPE and correlation coefficients r respectively.

As mentioned above, the proposed MetaBand enables multimodal outputs including the hot film output (HF) related to artery pulse and skin thermal conductivity, the cold film output (CF) related to forehead temperature, and the acceleration (Acc) related to human body activity. We propose a method of real‐time estimation of human total metabolic rate based on thermosensation via the multimodal physiological monitoring using the MetaBand. We adopt a CNN‐based neural network to map the MetaBand's multimodal outputs into the total metabolic rate, as shown in Figure 4A. To comprehensively extract the time‐frequency characteristics, we transfer the MetaBand outputs into the time‐frequency diagrams by STFT, including the raw outputs of the hot film (HF) and the cold film (CF) from the e‐skin, and the acceleration (Acc). The spectrogram window size, the STFT window size and the STFT step length is 10 s, 2 s and 1s respectively (Figure S11). The CNN model uses these time‐frequency diagrams as inputs and outputs the estimated total metabolic rate of human body in real time. The dependence of the estimated metabolic energy cost on different inputs during data fusion is analyzed (Figure S12). Among forehead temperature, head motion, and forehead skin thermal conductivity, the latter exhibits the dominant contribution. It indicates the strong correlation between energy cost and skin thermal conductivity. Besides, the best estimation performance occurs only when all the three inputs are used. Therefore, all the outputs of the MetaBand including HF, CF and Acc finally participate in metabolic estimation. The structure and hyperparameters of the CNN model are listed in Table S3.

The same typical daily exercise routine with that in Figure 3C is also used to validate the metabolic rate estimation using the MetaBand. The subject successively takes an initial rest, slow walking at 4 km/h, brisk walking at 6 km/h, light jogging at 7 km/h, intense running at 9 km/h, and a final rest. A medical respirometry system (Metalyzer 3B, CORTEX, Germany) is used for acquiring the reference of metabolic rate. 7 subjects participate the experiments (the ambient temperature and humidity 26.1 °C, 26% respectively). Four independent trials are conducted for each subject, of which 3 repetitions are for CNN model training and the remained repetition is for model test. Leave‐One‐Out cross validation is used, and the average performance is used for final analysis. The results of one trail of Sub. 1 are shown in Figure 4B. All the results of the subjects can be found in Figure 4C–D and Supplementary Materials (Figure S13–S19). As shown in Figure 4B, the metabolic rate monitored by the MetaBand increases with the locomotion intensity, and gradually returns to the initial state after the exercise. Besides, the total metabolic rate including both activity and resting energy expenditure is estimated. The average mean absolute percentage error (MAPE) of all the participants is 10.09% during the entire exercise routine (Figure 4C, and Table S4). The performance of total metabolic rate estimation among participants under different exercise intensity is also analyzed in Figure 4D. We can find that the estimation of MetaBand keeps consistent from static resting to intensive fast running, with the average absolute error (AAE) less than 0.50 W/kg (*N* = 7). The generalizability across the 7 subjects is also analyzed, as shown in Figure S20 and Table S5. Here, datasets from 6 subjects are used for model training, and the remained subject is used for test. The average result of four independent trials of each tested subject is used for analysis. The results show the average MAPE of 7 participants is 16.06% during the entire exercise routine. Therefore, our method exhibits good generalizability among people. Experimental results validate that the MetaBand is able to continuously and accurately estimate the total metabolic rate for both resting and active scenarios.

Figure 4E and Table S6 summarizes the comparison of multimodal physiological monitoring capability, including pulse waveform, heart rate, metabolic rate, and body temperature, between our method and existing wearable methods [14, 17, 19, 20, 24, 32, 48, 49, 50], for both static/steady and exercise situations. Here, Ste. HR and Exe. HR refer to heart rate measurements under steady and exercise activities respectively, Ste. Pulse and Exe. Pulse means pulse waveform captures under steady and exercise activities respectively, Sta. Meta and Exe. Meta refers to metabolic rate estimation under static and exercise situations respectively, Ste. BT and Exe. BT refer to body temperature measurements under steady and exercise activities respectively. The metrics of heart rate, pulse waveform, metabolic rate and body temperature used here are the reciprocal of AAE, pulse monitoring capability, the reciprocal of MAPE and correlation coefficients r respectively. It can be clearly seen that our method exhibits obvious advantages in both multimodality and accuracy of each modality, by only using a headband with compact sensor setup.

## 6 Continuous and Real‐time Applications for Personalized Health Management

Experiments are conducted to validate the feasibility of the MetaBand in continuous and real‐time monitoring of multimodal physiological parameters for personalized health management. During the experiments, the MetaBand records the sensor outputs, including the e‐skin and the accelerometer, and then transmits the data to a smart phone through Wi‐Fi. The smart phone figures out the forehead temperature, the pulse waveform, the total metabolic rate, and the stride, from the raw data, and displays the results on the screen in real time. The monitor gives warnings if the user is overfatigue or potential dangers (e.g., excessively high body temperature) exists during the exercise.

Before practical demonstrations, we evaluate the sweat influence on the MetaBand and the repeated usage performance. The standard ECG chest strap (Polar H10, Finland) and the medical respirometry system (Metalyzer 3B, CORTEX, Germany) are used for pulse waveform, heart rate, and metabolic rate references respectively. The sweating and non‐sweating experiments are designed as follows (the ambient temperature and humidity 25.6 °C, 13% respectively). One subject first conducts a non‐sweating trial including 5‐minute initial resting, 5‐minute 4 km/h slow walking, and 5‐minute post‐exercise resting. Then, the subject performs a warm‐up run at 7 km/h to achieve obvious sweating. Subsequently, the subject conducts a sweating trail suing the same setup with the non‐sweating trial. Results are shown in Figure S22 and Table S7. The MetaBand works consistently during both non‐sweating and sweating scenarios. Three repeated measurements of multimodal physiological information are also evaluated within 1 day. As shown in Figure S23, one subject performs three repeated exercise trials at 11:00 am (24.9 °C, humidity 16%), 4:00 pm (25.6 °C, humidity 13%), and 9:00 pm (24.9 °C, humidity 15%) in 1 day, respecitvely, inlcuding 5‐minute initial resting, 4‐minute 4 km/h walking, 4‐minute 7 km/h running, and 12‐minute post‐exercise resting. Results are shown in Figure S23 and Table S8. The MetaBand keeps working consistently during the 1‐day repeated tests.

**FIGURE 5:**
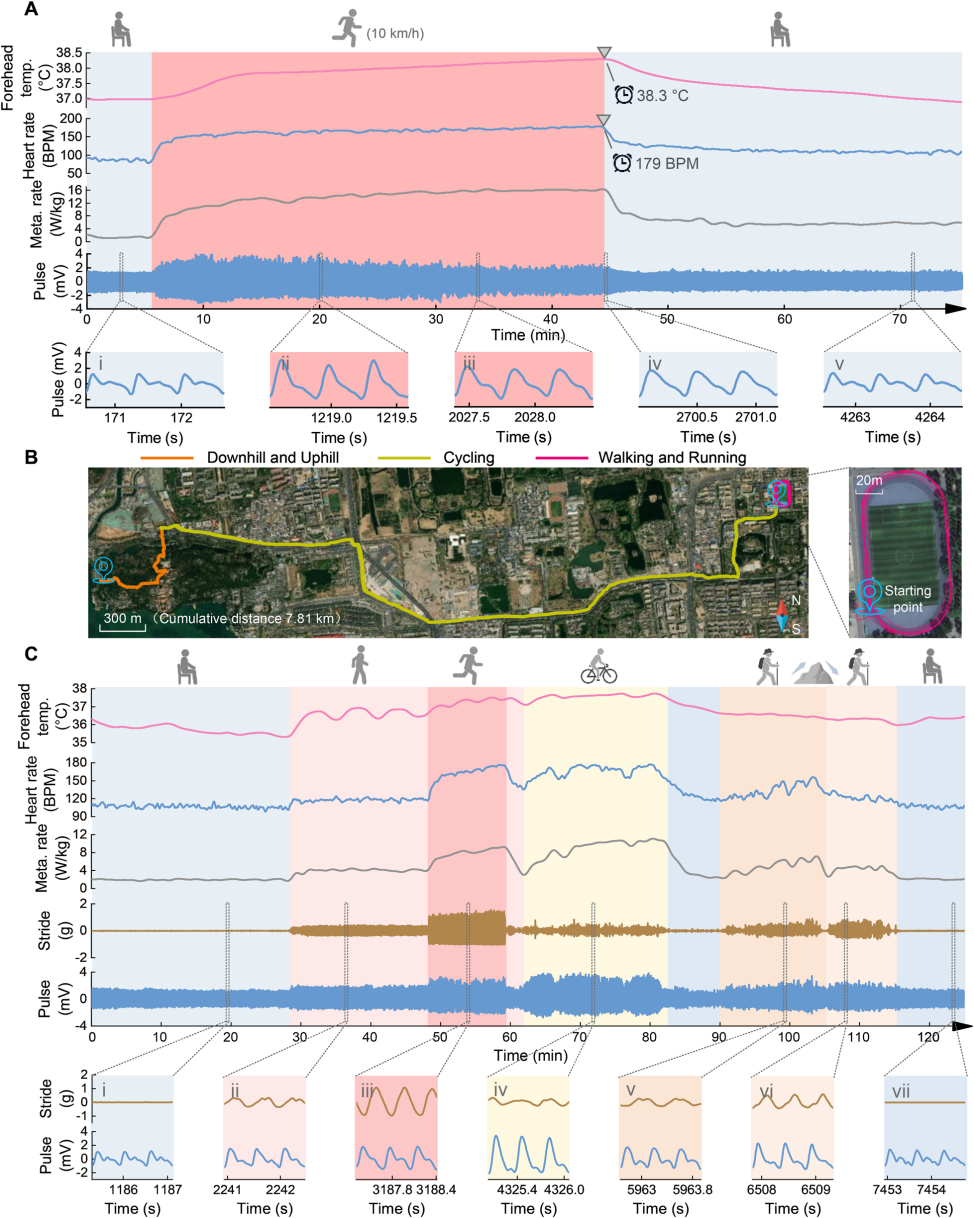
Results of continuous multimodal physiological monitoring during long‐term running and various outdoor activities. (A) Multimodal physiological monitoring during a long‐term running routine, including 40‐minute running at 10 km/h, 5‐minute pre‐exercise rest, and 30‐minute post‐exercise rest. The insets show detailed pulse waveforms during pre‐exercise rest, 15 minutes into running, 30 minutes into running, the moment running stops, and 25 minutes post‐exercise rest. (B) The trajectory of outdoor activities, including walking and running in a stadium, cycling on real roads, real uphill and downhill hiking, pre‐exercise and post‐exercise rest. The total accumulative distance of outdoor activities is 7.81 km. (C) Continuous multimodal physiological monitoring during various outdoor activities, including the pulse waveforms, total metabolic energy cost, heart rate, forehead temperature, and stride. The insets show detailed pulse waveforms and strides during pre‐exercise rest, outdoor walking, outdoor running, cycling, uphill and downhill hiking, and post‐exercise rest.

Then, we demonstrate the MetaBand's feasibility for long‐term continuous exercise with high intensity. A subject runs at 10 km/h for about 40 minute with the MetaBand worn on the head, and the multimodal physiological data are shown in Figure 5A (the ambient temperature and humidity 27.8 °C, 29% respectively). The forehead temperature increases with time, and reaches 38.3 °C after 40‐minute running, indicating the heatstroke risk if continuing high‐intensity exercise. The heart rate also increases with time while running, and reaches 179 BPM at the end of running. The total metabolic rate increases with time during the first 30 minutes of running, and gets steady (about 16 W/kg) during the last 10 minutes of running due to the balance of energy generation and consumption of human body. It is noted that, the total metabolic rate level, the heart rate and the forehead temperature are still kept obviously higher during the 30‐minute rest after long‐term running than those before running. This is the so‐called excess post‐exercise oxygen consumption (EPOC) phenomenon, because the body requires additional energy to recover to its pre‐exercise state, including replenishing energy stores, repairing muscle tissue, clearing lactic acid, and other recovery processes. The pulse waveforms are shown in Figure 5A, where the typical waveforms at various stages: before running, after 15 minutes of running, after 30 minutes of running, immediately upon stopping, and 25 minutes into post‐exercise rest, are shown in detail in the insets. The pulse waveform amplitude increases obviously at the beginning of the run compared to that during the pre‐exercise rest, and keeps high during the first 10 minutes of running. However, both the pulse waveform amplitude and the reflective peak gradually weakens during the last 30 minutes of running, indicating the increasing fatigue of human body during long‐term running. During post‐exercise rest, it takes over 20 minutes for the pulse waveform to return to its pre‐run state. The results are consistent with the fatigue index based on heart rate variability (HRV, Figure S21). Therefore, the pulse waveform can be used as an indicator of fatigue.

Besides, the pulse waveform contains rich information about cardiac load, cardiovascular condition, and potential risk. Comprehensive analysis on pulse waveform with different exercise intensities would provide a valuable tool for continuous and comprehensive investigation on cardiovascular disease pathogenesis.

The MetaBand is also demonstrated during a 2‐hour real‐world outdoor test, including various activities of walking and running in a stadium, cycling on real roads, real uphill and downhill hiking, pre‐exercise and post‐exercise rest, as shown in Figure 5 (the ambient temperature and humidity 17.0 °C, 37% respectively). The trajectory of outdoor activities is illustrated in Figure 5B, with the accumulative distance of 7.81 km. And the results (one subject) of multimodal physiological monitoring are shown in Figure 5C. The average speed of walking and running in the stadium is about 3.6 km/h and 6.5 km/h respectively. The average cycling speed is about 16 km/h, with a maximum speed about 21 km/h. The uphill and downhill hiking is conducted by naturally walking. The highest exercise intensity occurs around the 80th minute while the cycling, with the peaking of the forehead temperature, the heart rate, and the total metabolic rate simultaneously. During the hill climbing, the exercise intensity during the uphill walking is significantly higher than that during the downhill walking, even though the uphill walking speed is slower. This is evident from all physiological measurements, including forehead temperature, heart rate, and total metabolic rate. The pulse waveforms during different activities are shown in detail in the insets of Figure 5C. It can be seen that the pulse waveform changes with the activity and the intensity. The MetaBand works robustly during the outdoor test. In particular, the MetaBand provides accurate assessment of the total energy cost in real time, including the activity energy expenditure and the basic metabolic energy demand. The total energy consumption of the subject for the 2‐hour physiological and physical activities is about 618 kcal, which is equivalent to about 533 grams of steamed rice (116 kcal/100 gram) or 1.37 hamburger (450 kcal). The MetaBand is competent to provide the user scientific advices on eating and exercise to keep energy balance, achieving personalized health management.

## 7 Discussion

In this paper, we propose a thermal‐sensation‐based smart headband (MetaBand) for continuous monitoring of multimodal physiological parameters in human exercises, including pulse waveform, total metabolic rate, heart rate and forehead temperature. The MetaBand adopts a compact headband design for long‐term daily usage, on which a custom‐made e‐skin is integrated. The e‐skin is conformally attached on human skin over superficial temporal artery, and detects the forehead skin thermal conductivity, pulse waveform and forehead temperature simultaneously. The temporal has thin skin, low fat, and rich blood vessels, making it ideal for detection. Compared to trunk/limb‐mounted solutions, the head‐worn device experiences fewer motion artifacts due to the head's self‐stabilizing behavior and the damping effect of spine. What's more, an adaptive filtering method is proposed to further attenuate the residual motion artifact and ensures high‐fidelity acquisition of pulse waveform and heart rate. Adopting the MetaBand, a new method of estimating human total metabolic rate is proposed through comprehensively fusing the multimodal physiological parameters detected by the e‐skin and the activity intensity detected by an accelerometer. Eventually, the physiological parameters including the total metabolic rate, the pulse waveform, the heart rate and the forehead temperature are continuously and accurately monitored during human daily activities, even in high‐intensity exercises. Long‐term experiments containing various activities from resting, walking, jogging, bicycling, hill climbing and a long‐distance running, validate the effectiveness and robustness of the MetaBand. In particular, the pulse pressure waveform features varying with fatigue (e.g., the pulse waveform amplitude, the reflective peak, and the related timings) reveals rich information about cardiac load, cardiovascular condition, and potential risk. The MetaBand provides a promising tool for not only personalized sport training and daily health management, but also continuous and comprehensive investigation on cardiovascular disease pathogenesis.

## 8 Materials and Methods

### 8.1 Components of the MetaBand

Details of the design of the MetaBand are shown in Figure S1. The MetaBand consists of an elastic textile band, an e‐skin and a master unit. The band is made of 71.5% cotton, 21.7% polyester fiber, 5.99% elastic fiber and 0.9% spandex. The e‐skin is custom made, integrating two thin‐film thermosensitive sensors (Pt films in Figure 1A, named as the cold film and hot film respectively) on a flexible polyimide (PI) substrate. It is covered by a PDMS layer and then attached to the textile band using medical tape. The PDMS layer and the textile band provide thermal insulation for the e‐skin from the external environment. With such a thermal insulation setup, the e‐akin capably monitors the forehead temperature, the pule waveform and the total metabolic energy cost simultaneously, based on thermal sensation mechanism. All the related materials are flexible, ensuring conformal and comfortable attachment of the e‐skin onto human skin for reliable and robust continuous physiological monitoring. The master unit contains a conditioning circuit for the e‐skin and an Arduino module (Arduino Nano 33 IOT, Arduino S.r.l). The custom conditioning circuit connects with the e‐skin through shielded FPC (flexible printed circuit) wires and communicates with the Arduino module through a serial port. It is responsible for signal conditioning (Figure 2A) and analog‐to‐digital conversion of the e‐skin. The master unit contains a built‐in accelerometer, an edge computing module, and a Wi‐Fi module. The built‐in accelerometer is used to monitor human body activity. The Arduino module collects data from the built‐in accelerometer and the e‐skin at 100 Hz, and then transmits the raw data to a smart phone through Wi‐Fi in real time. An app is developed and runs on the smart phone for real‐time data processing, display and storage. We use Wi‐Fi for demonstration because of its long transmission distance and stability. This is important for high‐quality data transmission especially during the outdoor tests. Our MetaBand's power consumption is about 528 mW, where the e‐skin is about 9 mW, the master unit including the conditioning circuit of e‐skin, a built‐in accelerometer, an edge computing module, and a Wi‐Fi module is about 519 mW. By using a 300 mAh internal battery, the MetaBand is able to work continuously for more than 2 hours.

### 8.2 Fabrication of the e‐skin

The e‐skin consists of a sensing layer and a PDMS thermal‐insulation layer, and is fabricated by the following process (Figure S2): (i) A commercial flexible polyimide substrate (AP8525R, DuPont Co. Ltd., Wilmington, America) is prepared for the fabrication of the sensing layer. (ii) Pads and wires are fabricated in advance on the polyimide substrate by flexible‐printed‐circuit‐board (FPCB) fabrication process. (iii) The shape of cold and hot films is patterned by spin coating and then lithography of 30 µm thick photoresist (KXN5735‐LO, Rdmicro Co. Ltd., Suzhou, China) on the polyimide substrate. (iv) The metal layer of cold and hot films is deposited by sequentially sputtering chromium/platinum (35 nm/140 nm) onto the patterned substrate. (v) Patterned cold and hot films are formed after 2 h soaking in acetone to remove the photoresist. (vi) A protective layer of parylene with a thickness of 6 µm is deposited to provide electrical insulation from the skin and to prevent sweat pollution as well. (vii) A pure PDMS layer (the ratio of base agent: cross‐linker was 10:1 wt%) is cured in a PMMA mold (Φ10 mm× 2 mm) at 75°C for 2 hours. (viii) After demolding and drying, adhere the PDMS layer to the bottom side of the sensing layer.

### 8.3 Parameter Recognition for Motion Artifact Model Based on Bayesian Optimization

Bayesian optimization has been proved effective and efficient for global optimization of multiple parameters, especially for scenarios with objective functions lacking analytic derivatives or are expensive to calculate. Here, we use the average similarity between the captured pulse waveforms during the calibration test (2‐mininute natural running) as the objective function, which is scaled by the normalized Euclidian distance. It is noted that, the time sequences of pulse waveforms are normalized into the same length in advance. The normalized Euclidian distance is shown in equation (2), where **P**_*i*_ is the *i*th pulse waveform, *n* is normalized length, and d_Euc_(·) refers to the Euclidian distance between two vectors. For efficient computation, we randomly select 20 pulse waveforms to calculate their Euclidian distances to all the pulse waveforms and give the output of the objective function *f*(**x**) using the average distance, as shown in equation (3), where *N* is the total number of captured pulse waveforms, **x**  =  [*K*,  ζ,  ω_*n*_] is the vector of model parameters need to be recognized.

(2) $$D\;\left(i,j\right)=\frac{d_{\mathrm{Euc}}\left(P_{i}\;\cdot \;P_{j}\right)}{n}\;$$

(3) $$f\;\left(x\right)=\frac{\sum_{i=1}^{20}\sum_{j=1}^{N}D\left(i,j\right)}{20\times N}$$

Expected improvement (EI) is selected as the acquisition function, which naturally balances exploration and exploitation. The Bayesian optimization is initialized with 8 random selected vectors of **x** to give the initial prior distribution knowledge of *f*(**x**). After initial evaluation, Bayesian optimization estimates the posterior distribution while checking a new **x** based on the prior knowledge, and selects the new **x** as the next point in the search space by maximizing EI. Here, *f*(**x**) is modeled using a Gaussian process with Gaussian noise $\varepsilon ∼N(0,\;\sigma_{n}^{2})$. At the *t*th iteration of optimization, a dataset of model parameters and the corresponding average Euclidian distance between pulse waveforms is attained, **D**  =  {**X**, **y**}, where $X={\left[x_{1},x_{2},\text{…},x_{t}\right]}^{T}\;\in R^{t\times 2N}$, $y={\left[y_{1},y_{2},\text{…},y_{t}\right]}^{T}\;\in R^{t}$, y_*t*_ =  *f*(**x**_*t*_). The prior distribution can be described by mean *m*(**X**) and covariance K(**X**, **X**). Without loss of generality, *m* (**X**) =  0. And K_*ij*_ =  k(**x**_*i*_,**x**_*j*_) uses ARD Matern5/2 as the kernel function. For a new point to be checked x_*_, the posterior distribution of *f*(x_*_) can be calculated based on the prior distribution and the dataset **D**. The mean and variance of the posterior at x_*_ can be derived from (4) and (5) respectively, where k_*_ = * *[k(**x**_1_,x_*_),⋅⋅⋅, k(**x**_*t*_,x_*_)].

(4) $$\mu \left(x_{*}\right)=k_{*}^{T}\;{\left(K+\sigma_{n}^{2}I\right)}^{-1}y$$

(5) $$\sigma^{2}\;\left(x_{*}\right)=k\left(x_{*},x_{*}\right)-k_{*}^{T}{\left(K+\sigma_{n}^{2}I\right)}^{-1}k_{*}$$

Then x_*_ is selected as the next point **x**_*t* + 1_ by maximizing EI, which is the expected reduction in average Euclidian distance over the smallest value observed previously, $\mathrm{EI}\;\left(x_{*}\right)=\max\left(f_{\min}-f\left(x_{*}\right),0\right)$. As shown in equation (6), x_*_ is determined by the equivalent form of EI, where χ is the search space of **x**, *f*_min_ = min_*i* = 1, ⋅⋅⋅, *t*_ E[*f*(**x**_*i*_)], Φ(·) and ϕ(·) are the cumulative distribution function and probability density function of the normal distribution, respectively.

(6) $$\begin{array}{ccc} & & x_{*}=\mathrm{argma}x_{x_{*}\in \chi }\;\left(f_{min}-\mu \left(x_{*}\right)\right)\Phi \left(\frac{f_{min}-\mu \left(x_{*}\right)}{\sigma \left(x_{*}\right)}\right)\\ & & +\sigma \left(x_{*}\right)\phi \left(\frac{f_{min}-\mu \left(x_{*}\right)}{\sigma \left(x_{*}\right)}\right) \end{array}$$

### 8.4 Neural Network Model for Metabolic Estimation

Deep learning has provided a powerful tool for data analysis. Here, we propose to use CNN‐based neural network for analysis of the metabolic energy cost dependence on physiological activity intensity, heat state and physical activity intensity implied from the MetaBand outputs. And finally, a CNN‐based neural network model is established to map the MetaBand's multimodal outputs into the total metabolic rate, as shown in Figure 4A. To comprehensively extract the time‐frequency characteristics, we transfer the MetaBand outputs into the time‐frequency diagrams by STFT, including the hot film output (HF) related to skin thermal conductivity and artery pulse, the cold film output (CF) related to forehead temperature, and the acceleration (Acc) related to human body activity. The spectrogram window size, the STFT window size and the STFT step length is optimized as 10 s, 2 s and 1s respectively (Figure S11). The CNN model uses these time‐frequency diagrams as inputs and outputs the estimated metabolic rate of human body in real time. The TensorFlow framework (TensorFlow 2.10) including the built‐in Keras framework is used for network modeling, and the Adam optimizer is adopted for model training. Leave‐One‐Out cross validation is utilized for network model evaluation, where 3 of the 4 independent repeated trials are used for CNN model training and the remained repetition is used for model test. The average network model performance of each cross‐validation setup is used in the final evaluation. The hyperparameter optimization of STFT is shown in Figure S11, and the optimal structure and parameters for layers of the neural network model are shown in Table S3 in detail.

### 8.5 Participants

Subjects were given the necessary background information about the experiment and what tasks they should expect to complete. All were healthy and had no medical conditions which would interfere with their ability to do exercise. Informed consent was obtained from the human subjects to use their image and conduct the experiments described in this paper. The experiments performed for this study involving human subjects were approved by the Institution Review Board of Tsinghua University (No. 20180009).

### 8.6 Statistics

Statistical analyses were conducted using Matlab 2024a (MathWorks, USA).

## Author contributions

S.L. and G.C. contribute equally to this work. S.L. and R.Z. conceptualized the method. S.L., J.Y. and G.C. designed and fabricated the system. S.L. and J.P. designed the software. S.L. and G.C. carried out data analysis. S.T. and Q.M. fabricated the e‐skin. G.C and C.W. test the sensors. S.L. and R.Z. were the supervisor of the research. S.L. and R.Z. co‐wrote the manuscript.

## Funding

This work is supported by the Beijing Natural Science Foundation (Grant No. L247001), the National Natural Science Foundation of China (Grant No. 51735007, No. 62003184 and No. 62401333), the Postdoctoral Science Foundation of China (Grant No. 2023M742014).

## Conflicts of Interest

The authors declare no conflicts of interest

## Data and Availability Statement

All data are available in the main text or the supplementary materials. The data that support the findings of this study are available from the corresponding author upon reasonable request.

## Supporting information




**Supporting File 1**: advs74279‐sup‐0001‐SuppMat.docx.

**Supporting File 2**: advs74279‐sup‐0002‐MovieS1.mp4.

**Supporting File 3**: advs74279‐sup‐0003‐MovieS2.mp4.
