# Supplementary material for: A Smart Headband for Multimodal Physiological Monitoring in Human Exercises
Source: Adv Sci (Weinh). 2026 Feb 8;13(22):e74279. doi: 10.1002/advs.74279 (PMC13088344; doi:10.1002/advs.74279)
Supplement: Supplementary file 1 — Supporting File 1: advs74279‐sup‐0001‐SuppMat.docx. [file ADVS-13-e74279-s001.docx]

Supporting Information for

**A smart headband for multimodal physiological monitoring in human exercises**

Shiqiang Liu *et al.*

*Corresponding author. Email: liushiqiang@mail.tsinghua.edu.cn, zr_gloria@mail.tsinghua.edu.cn

**This PDF file includes:**

Figs. S1 to S23

Tables S1 to S8

**Other Supplementary Materials for this manuscript include the following:**

Movies S1 to S2


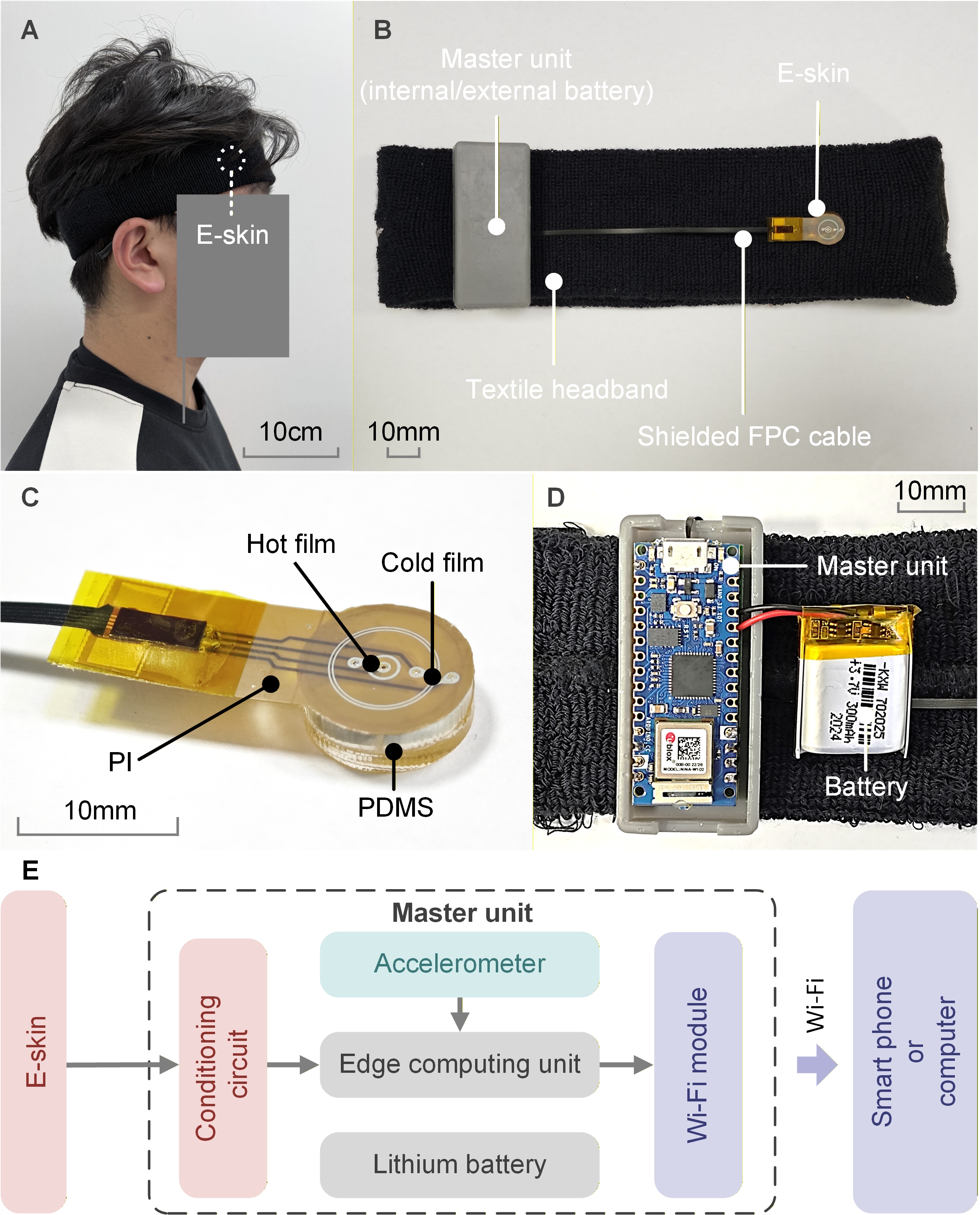


Fig. S1. Design of the MetaBand.

(**A**) MetaBand worn on a user. (**B**) Components and wire connections of the system. (E-skin weighs 0.60g, the master unit weighs 24.41g, and the MetaBand weighs 67.40g.) (**C**) Side view of e-skin. (**D**) Top view of the master unit. (**E**) The schematic diagram of MetaBand.


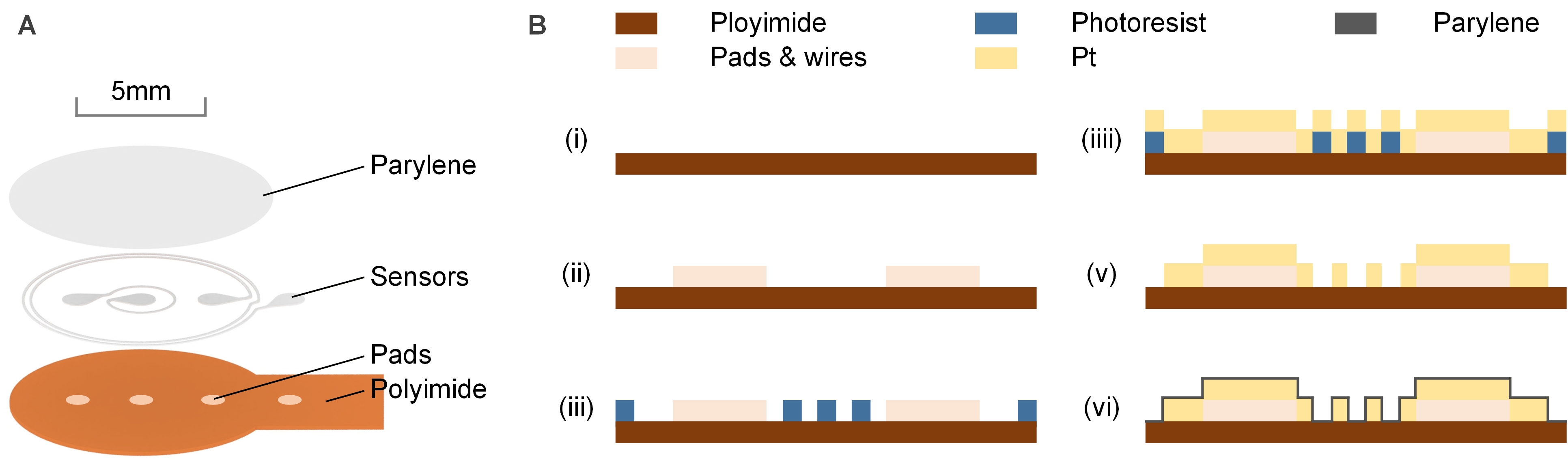


Fig. S2. Fabrication process of the e-skin.

(**A**) The structure of the e-skin. (**B**) Fabrication process of the e-skin based on flexible-printed-circuit and MEMS technology.


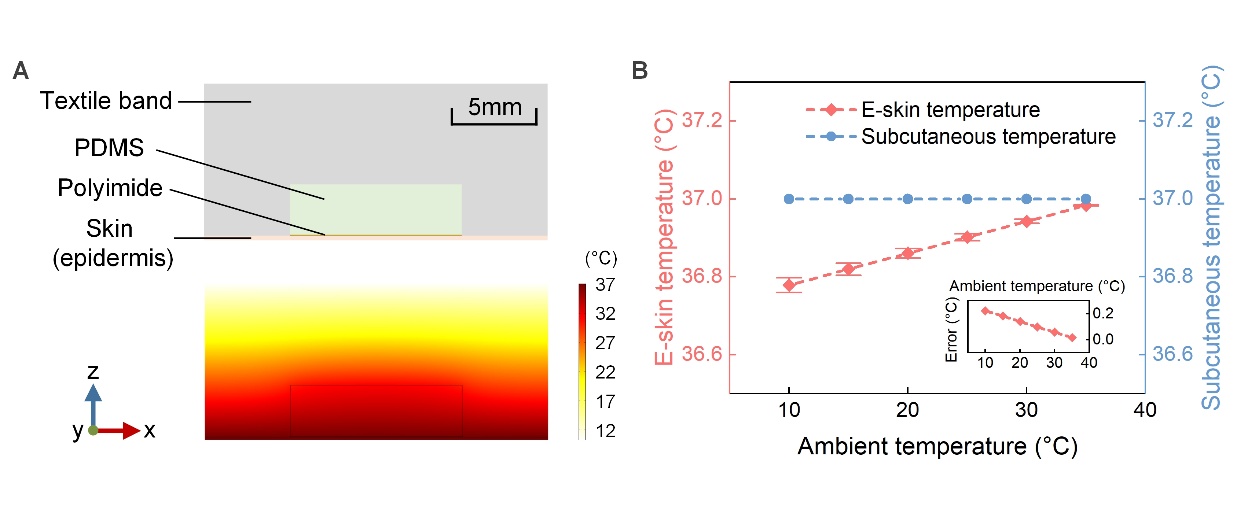


Fig. S3. Simulation results of the thermal insulating layers.

(**A**) FEM simulation results obtained through COMSOL. The model structure from bottom to top is skin, polyimide, PDMS and textile band respectively. (**B**) Simulation results of E-skin layer under the influence of different ambient temperatures and constant subcutaneous temperatures.


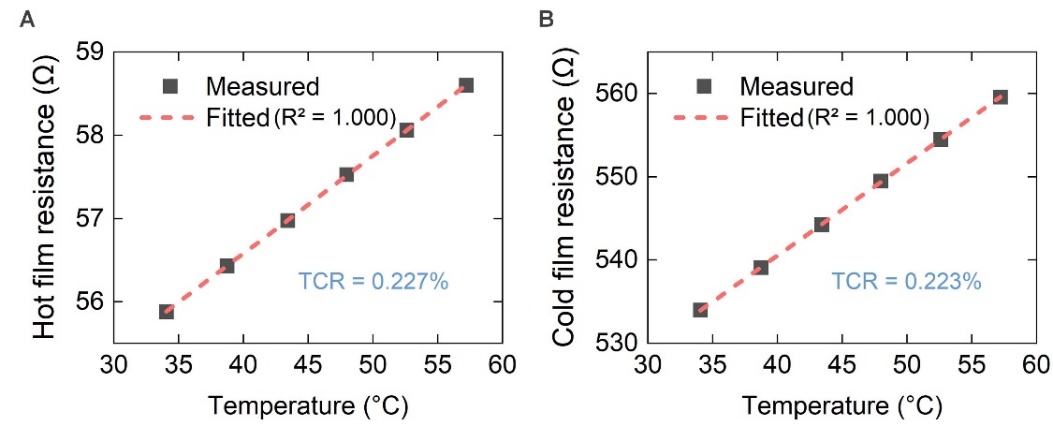


**Fig. S4. Thermal sensing charactaristics of the hot film and cold fim sensors in the e-skin.**

(**A**) Temperature response of the hot film sensor and the temperature coefficient of ressitance (TCR). (**B**) Temperature response of the cold film sensor and the temperature coefficient of ressitance (TCR).


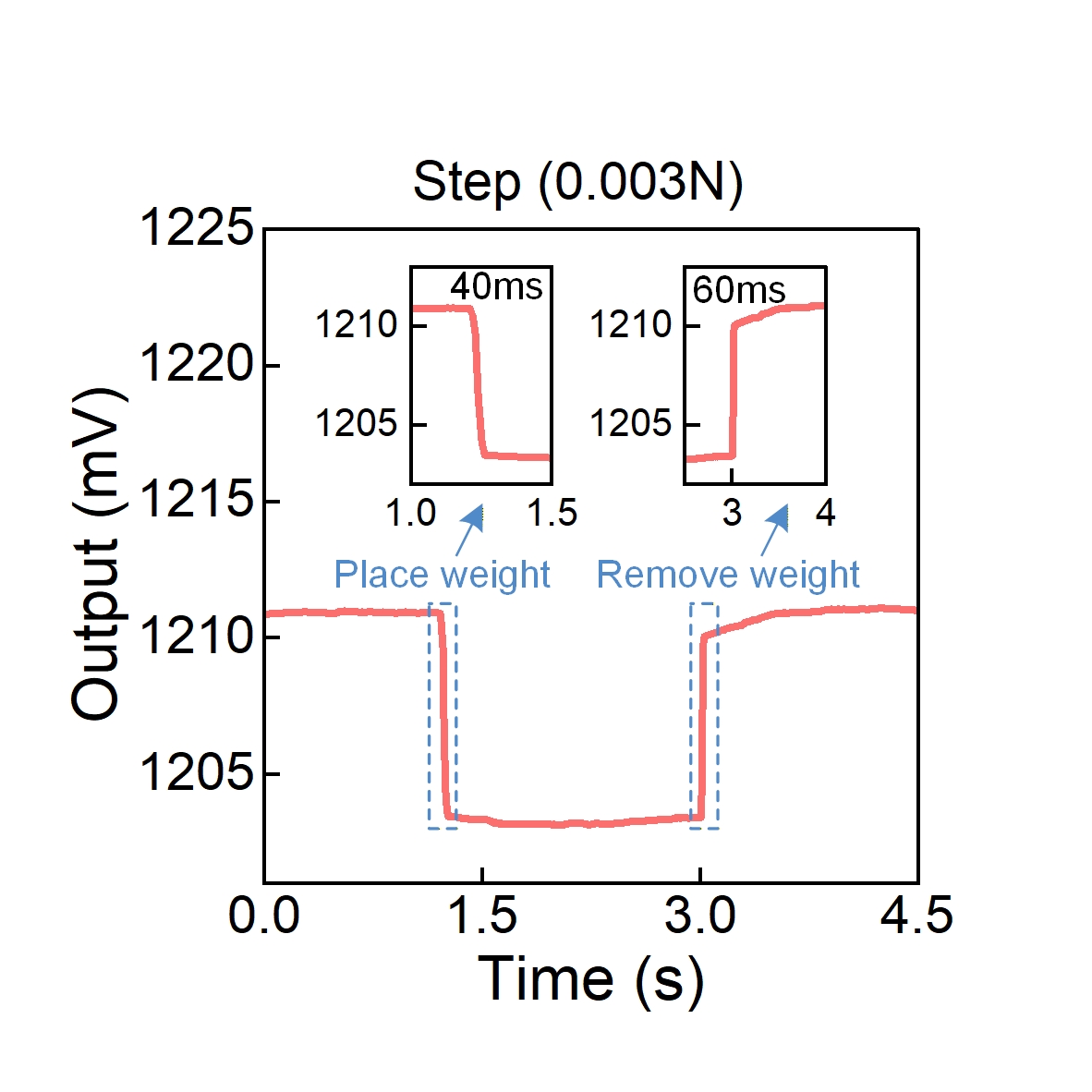


**Fig. S5. Dynamic response of hot film.**

The dynamic response of the sensor is tested by placing a tiny cap nut (0.3 g) on the sensor covered with a PDMS mimicking human skin and recorded in real-time. The response time for placing weight is 40ms, and the response time for removing weight is 60ms.


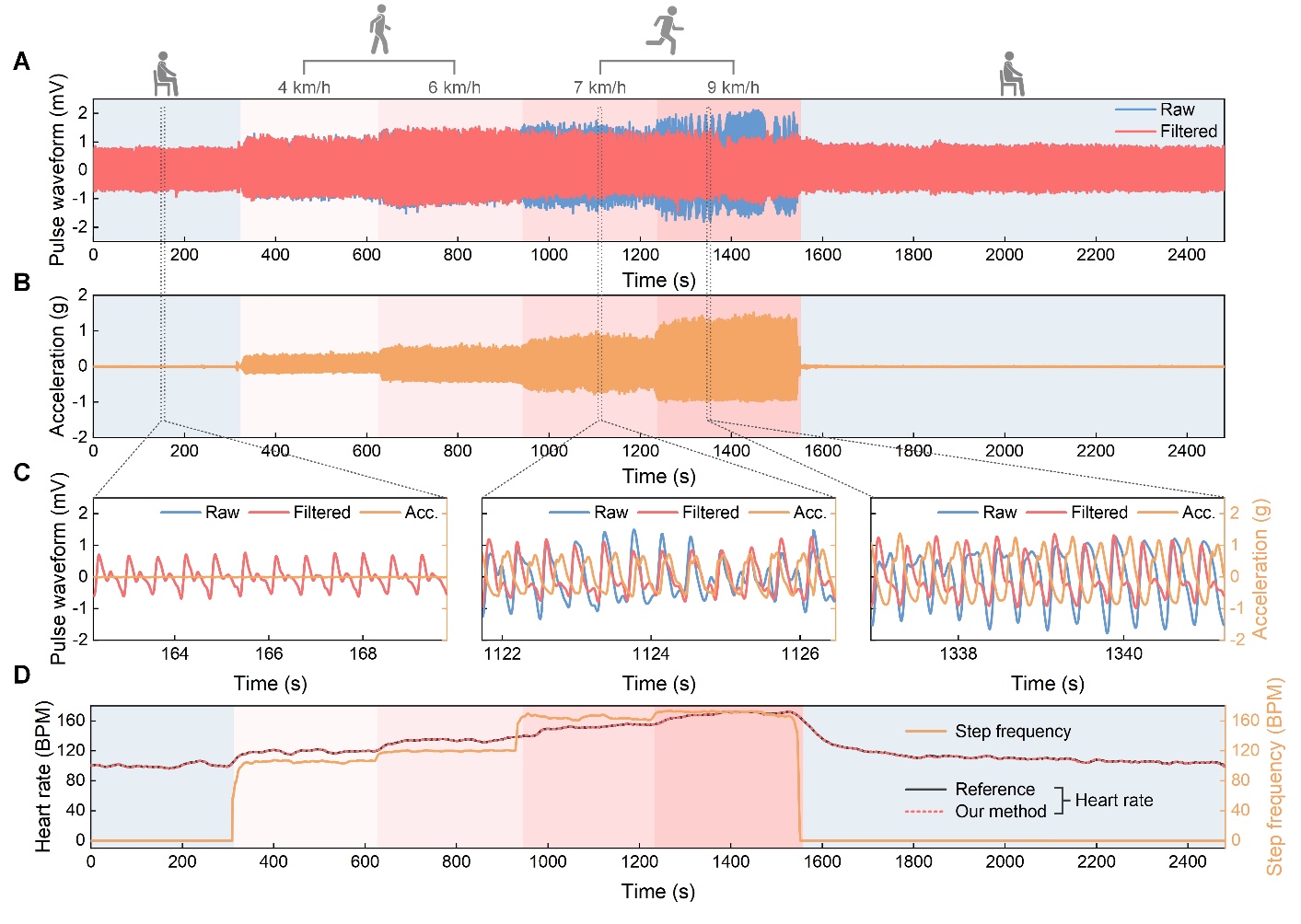


**Fig. S6. Comparison of raw and filtered pulse waveform, and acceleration signal.**

(A) The raw and motion-artifact-removed (filtered) pulse waveforms during the mimicked exercise routine. (B) The acceleration signal waveform during the mimicked exercise routine. (C) Insets show the pulse waveforms and acceleration signal in detail while resting and running. (D) Heart rate extracted from filtered pulse signal and step frequency extracted from acceleration signal.


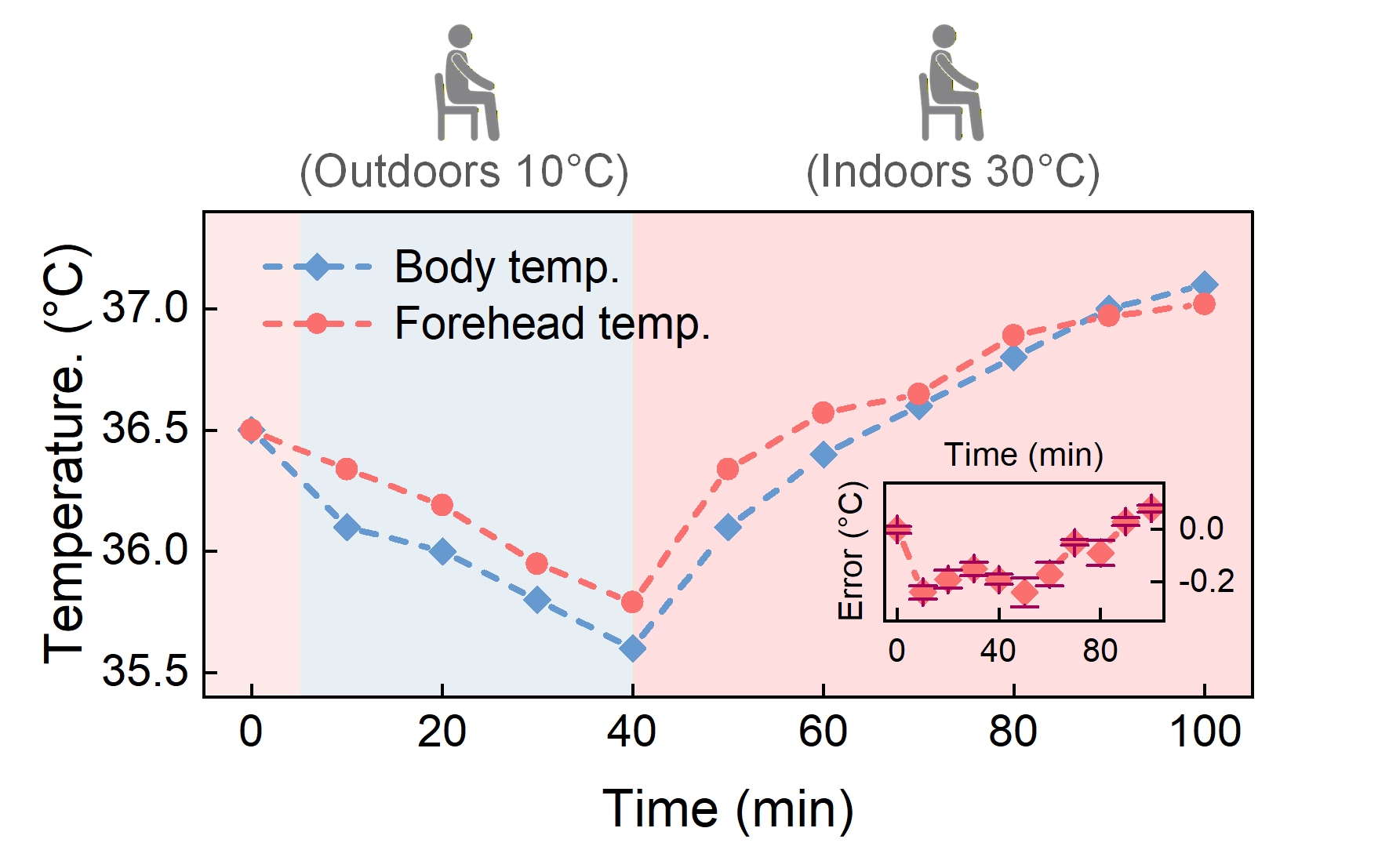


**Fig. S7. Temperature measurement under real-world outdoor and indoor conditions.**

The temperature measurement is started indoor (25 °C), and then is tested in the real world outdoor (10 °C, with natural airflow) for about half an hour, and indoor (30 °C) for an hour.


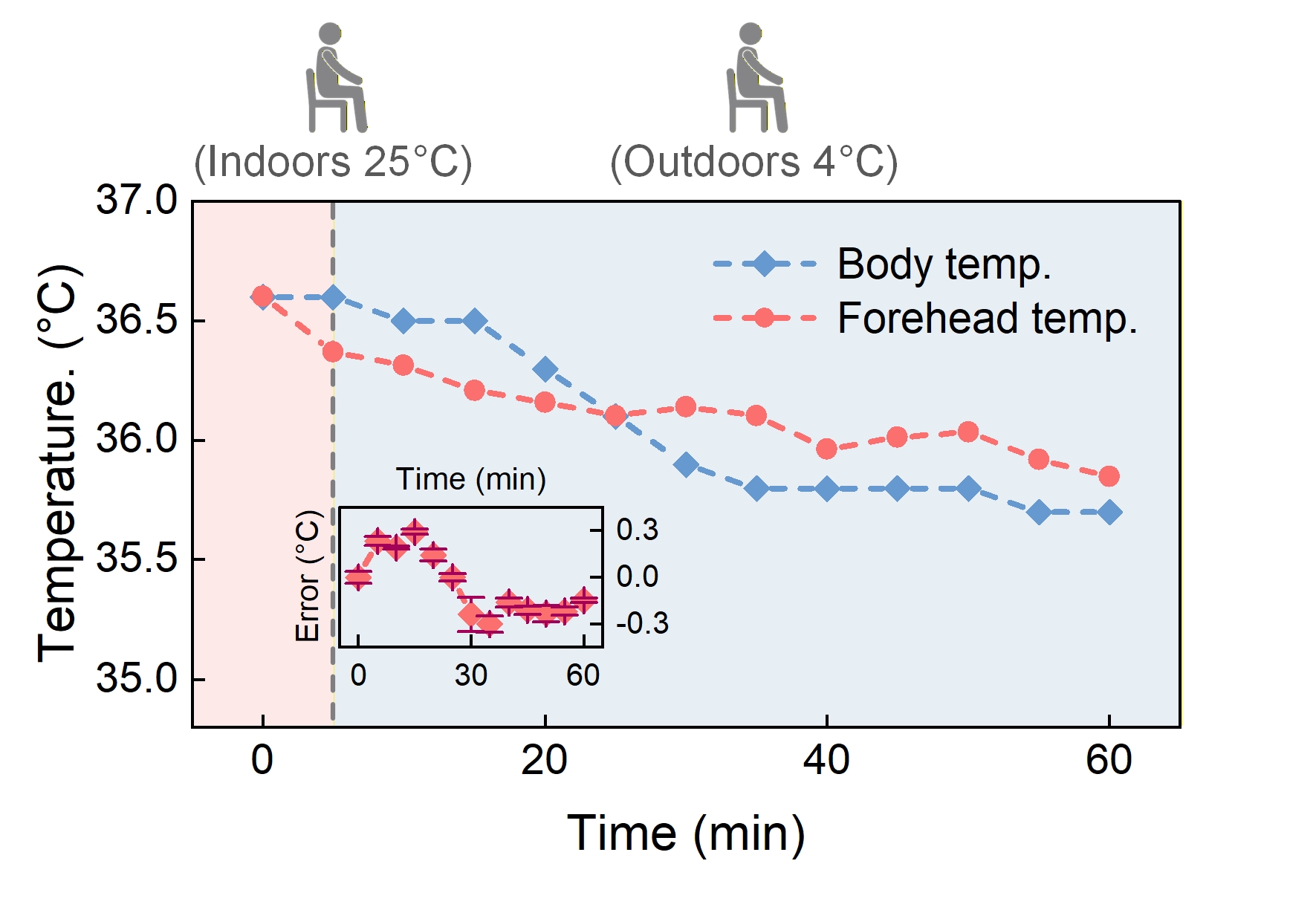


**Fig. S8. Temperature measurement under 4°C real-world outdoor conditions.**

The temperature measurement is started indoor (25 °C), and then is tested in the real world outdoor scenario (4°C, with natural airflow) for about an hour. The deviation is less than

0.30°C.


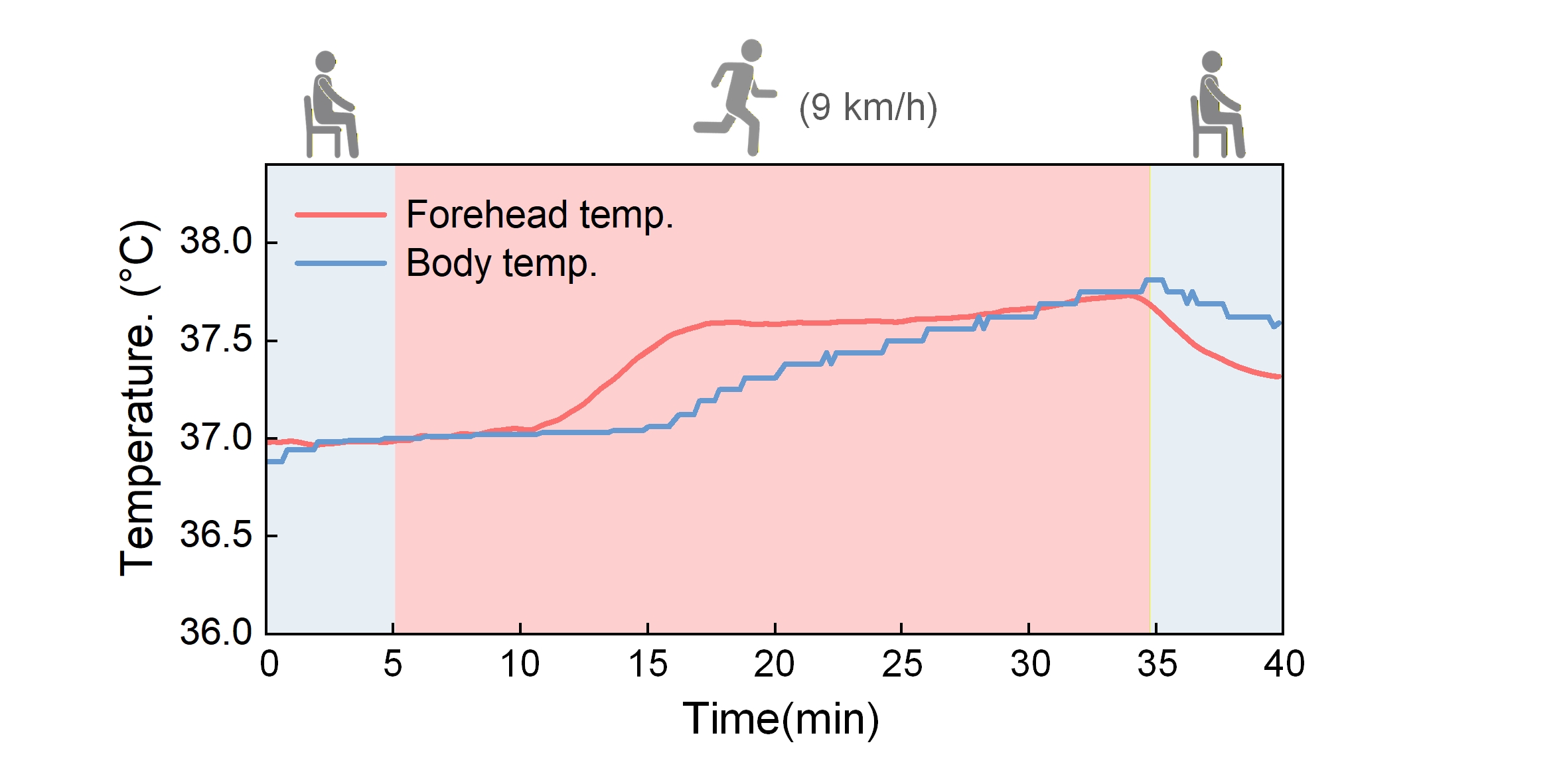


**Fig. S9. Temperature measurement during high-intensity running（Sub. 2）**

The exercise experiments include 5-minute initial rest, 30-minute intense running at 9 km/h, and 5-minute final rest.


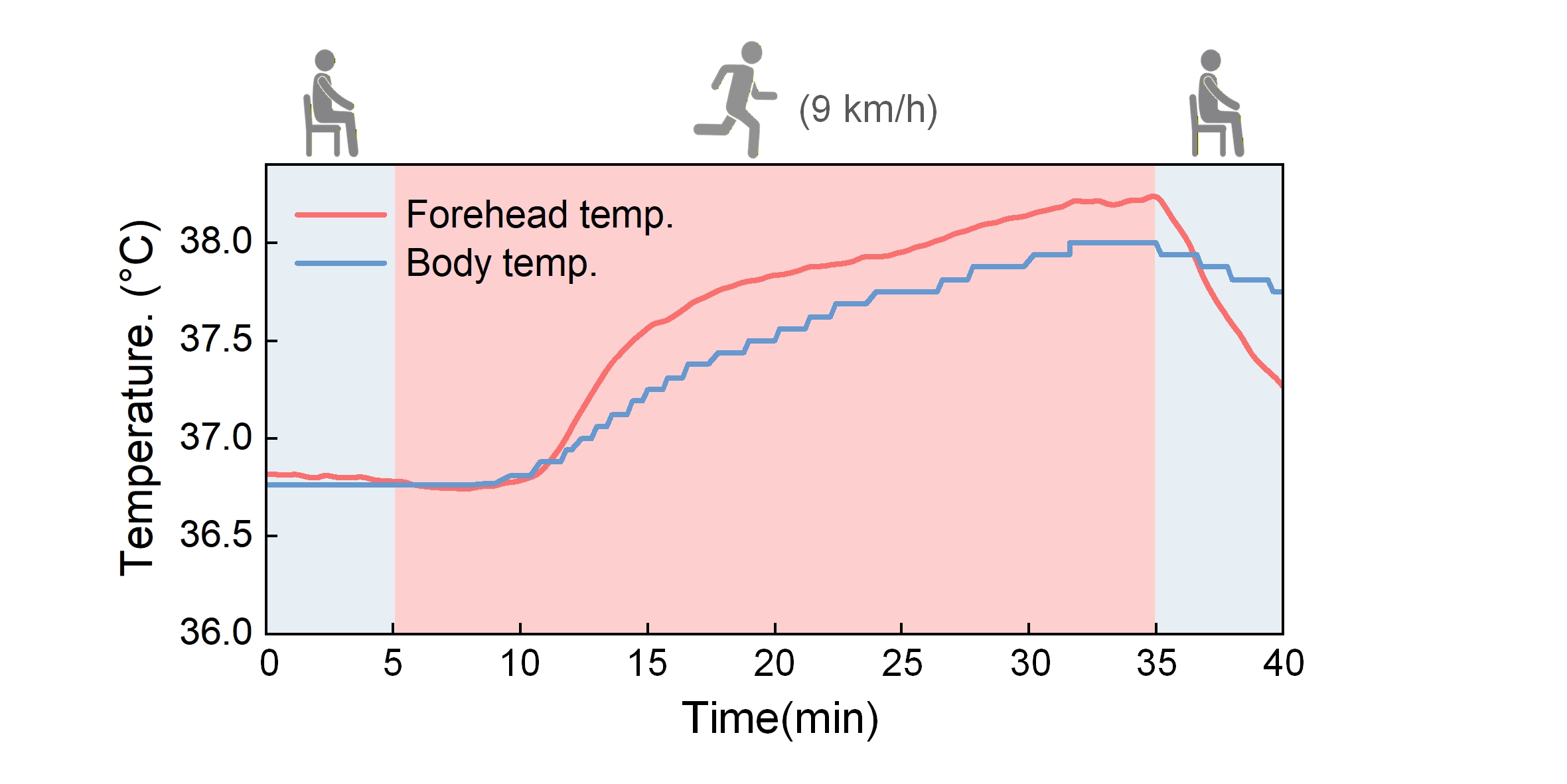


**Fig. S10. Temperature measurement during high-intensity running（Sub. 3）.**

The exercise experiments include 5-minute initial rest, 30-minute intense running at 9 km/h, and 5-minute final rest.


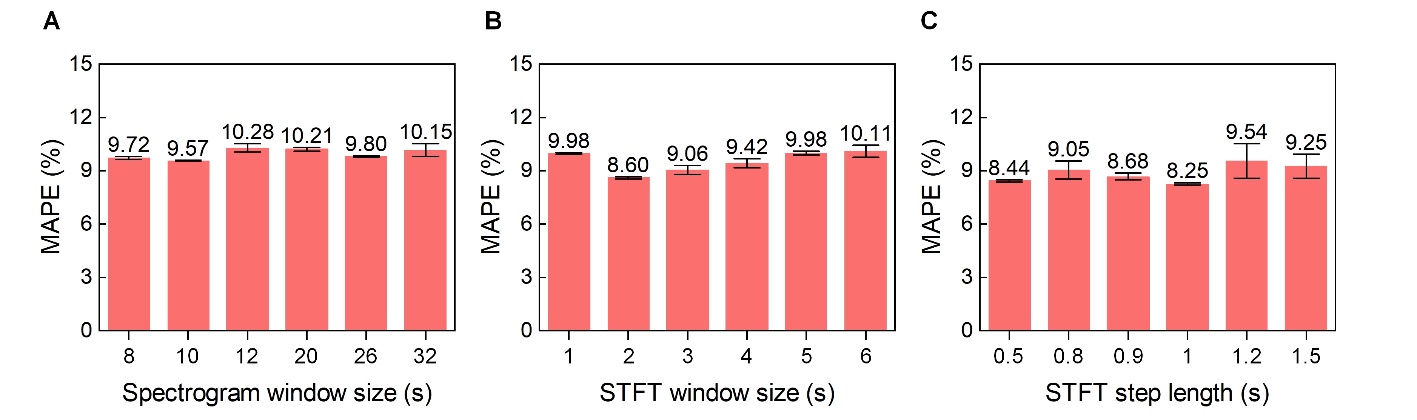


**Fig. S11. Optimization results of STFT parameter.**

(**A**) The metabolic rate estimation performance versus spectrogram window size. (**B**) The metabolic rate estimation performance versus STFT window size. (**C**) The metabolic rate estimation performance versus STFT step length.


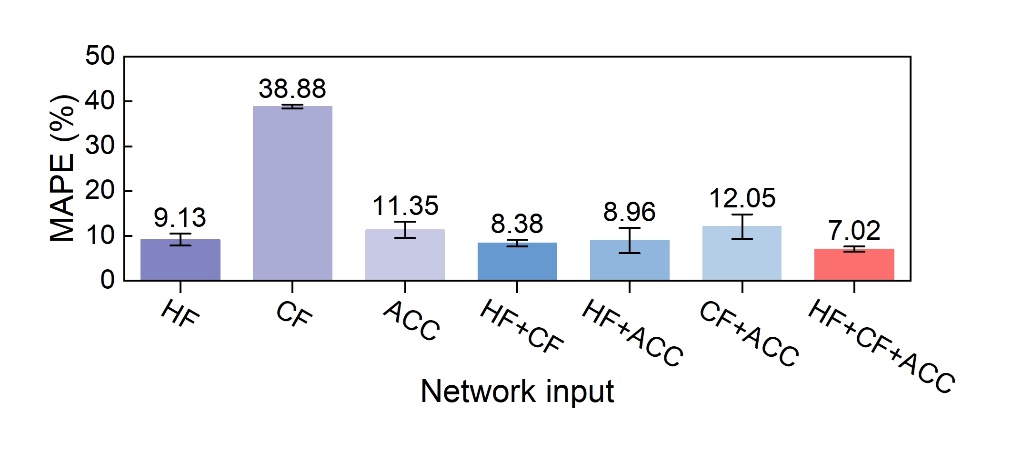


**Fig. S12. The performance while using different information for total metabolic energy cost estimation (Sub. 1).**

The average result of four independent trials of subject 1 is used for analysis. The error bar refers to the standard deviation.

**
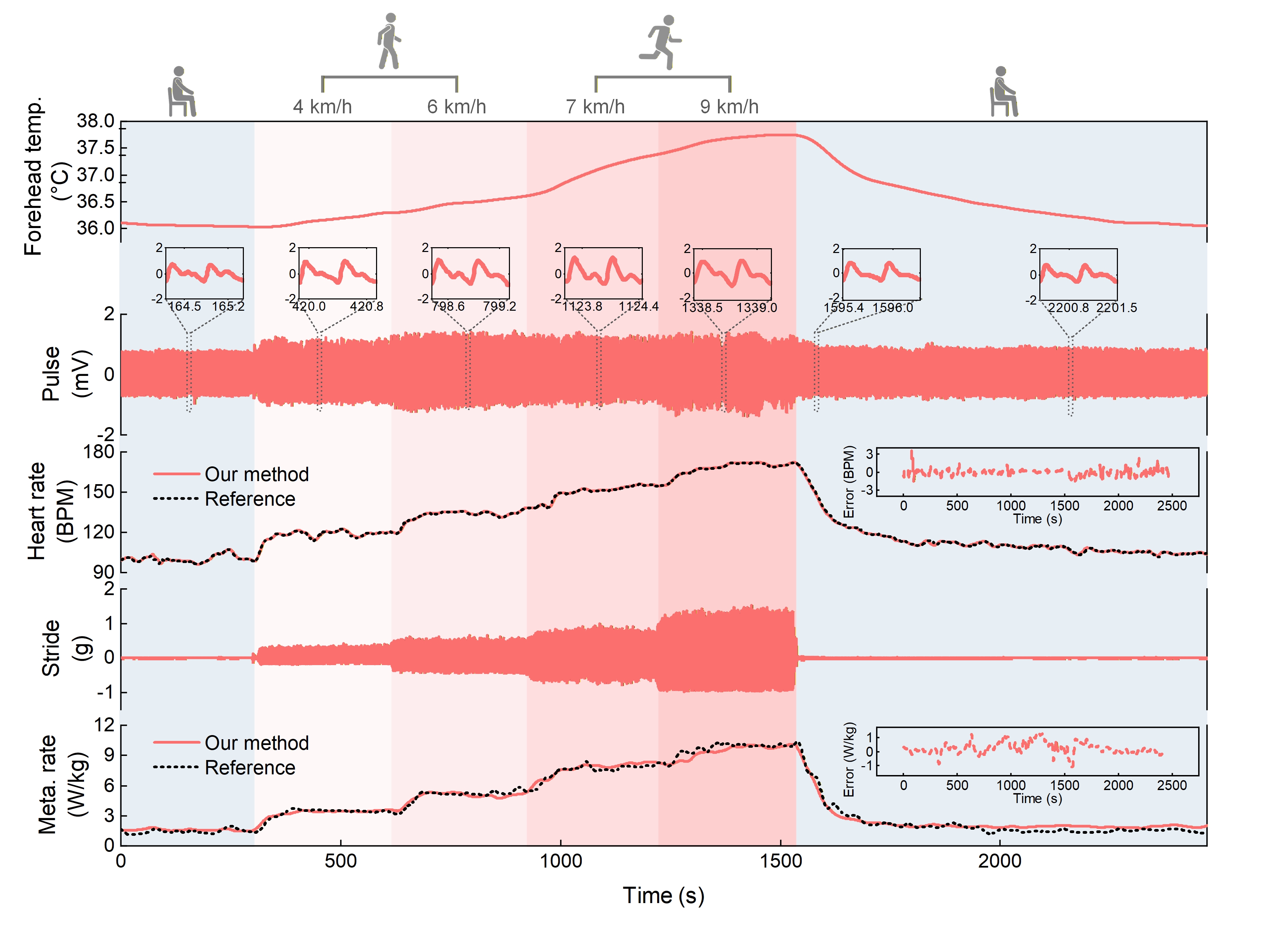
**

**Fig. S13. Results of multimodal physiological monitoring (Sub. 1).**

The insets show the detailed pulse waveforms of resting before exercise, slow walking, fast walking, slow running, fast running, stopping time of running, and resting after exercise. Meta. rate refers to the total metabolic energy cost of the paticipant per kilogram of body weight and per hour.

**
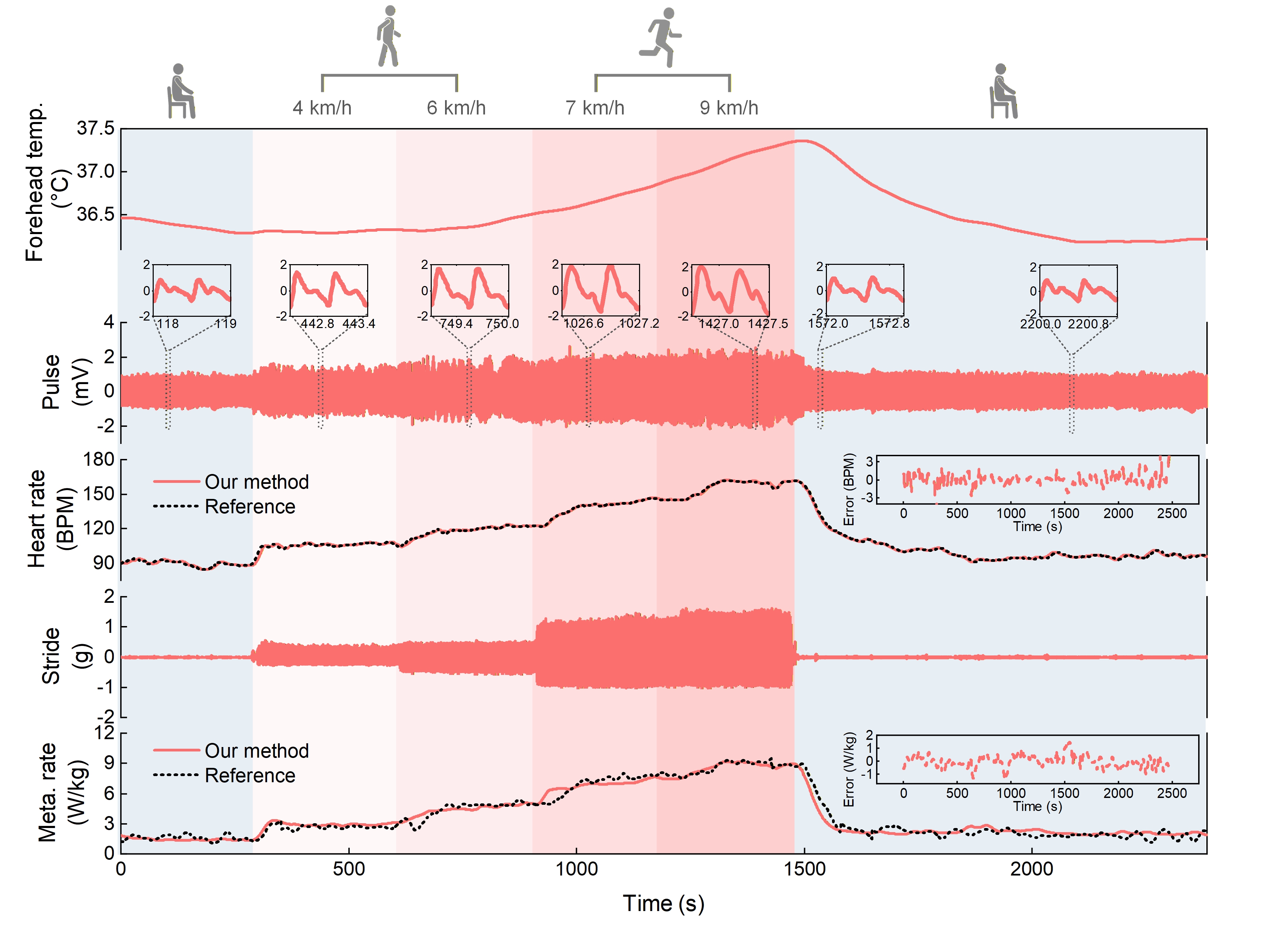
**

**Fig. S14. Results of multimodal physiological monitoring (Sub. 2).**

The insets show the detailed pulse waveforms of resting before exercise, slow walking, fast walking, slow running, fast running, stopping time of running, and resting after exercise. Meta. rate refers to the total metabolic energy cost of the paticipant per kilogram of body weight and per hour.

**
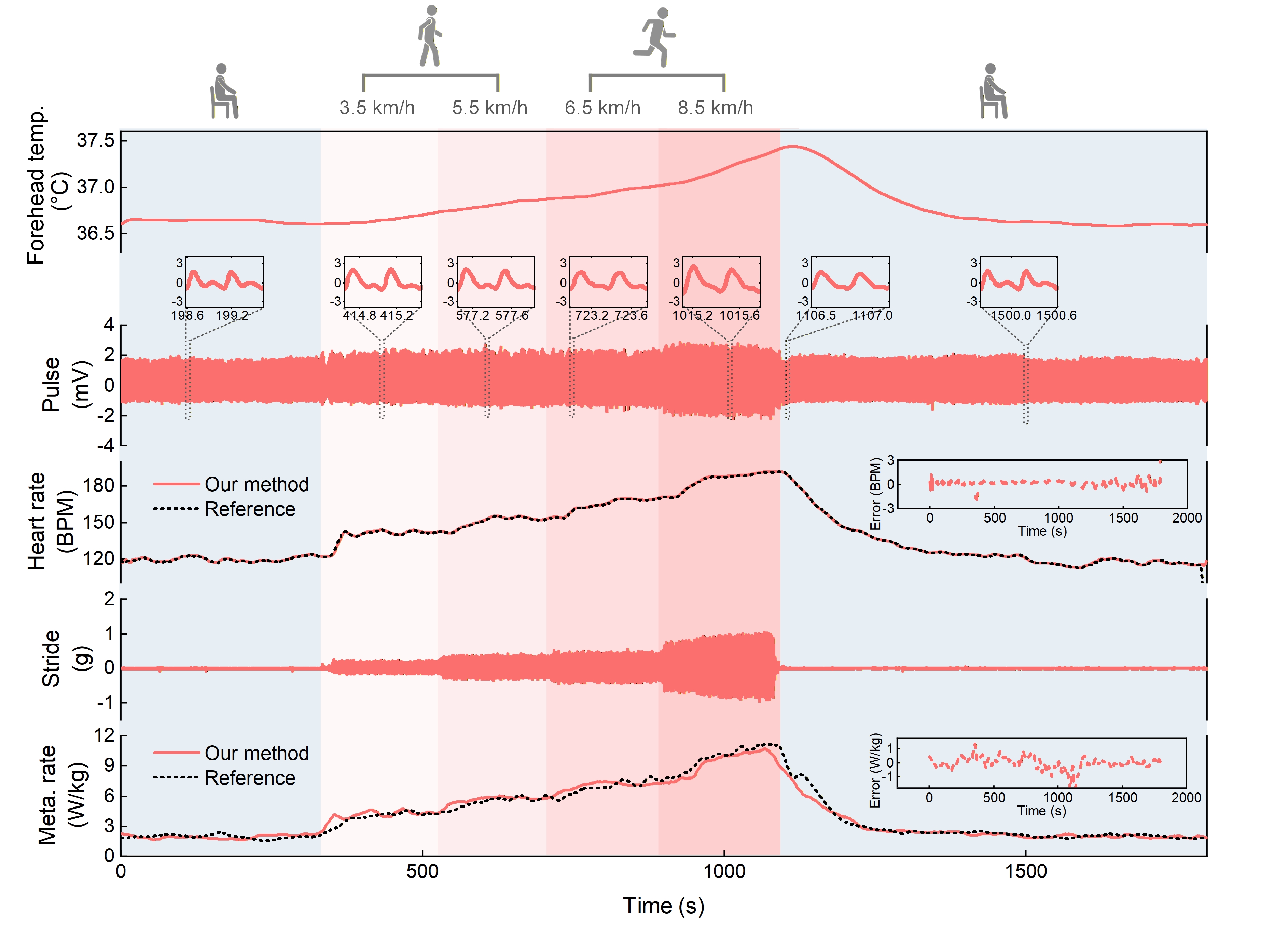
**

**Fig. S15. Results of multimodal physiological monitoring (Sub. 3).**

The insets show the detailed pulse waveforms of resting before exercise, slow walking, fast walking, slow running, fast running, stopping time of running, and resting after exercise. Meta. rate refers to the total metabolic energy cost of the paticipant per kilogram of body weight and per hour.

**
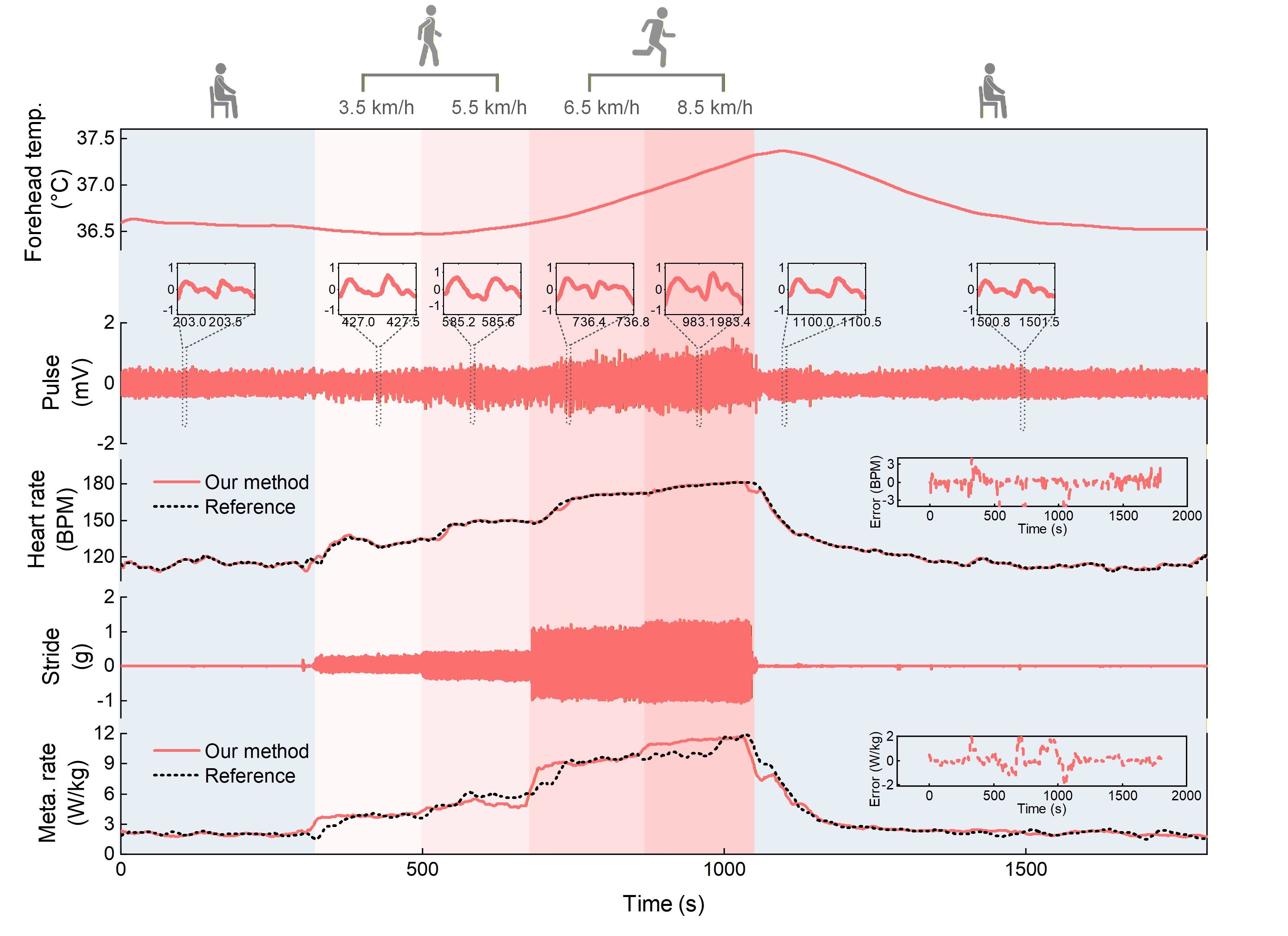
**

**Fig. S16. Results of multimodal physiological monitoring (Sub. 4).**

The insets show the detailed pulse waveforms of resting before exercise, slow walking, fast walking, slow running, fast running, stopping time of running, and resting after exercise. Meta. rate refers to the total metabolic energy cost of the paticipant per kilogram of body weight and per hour.

**
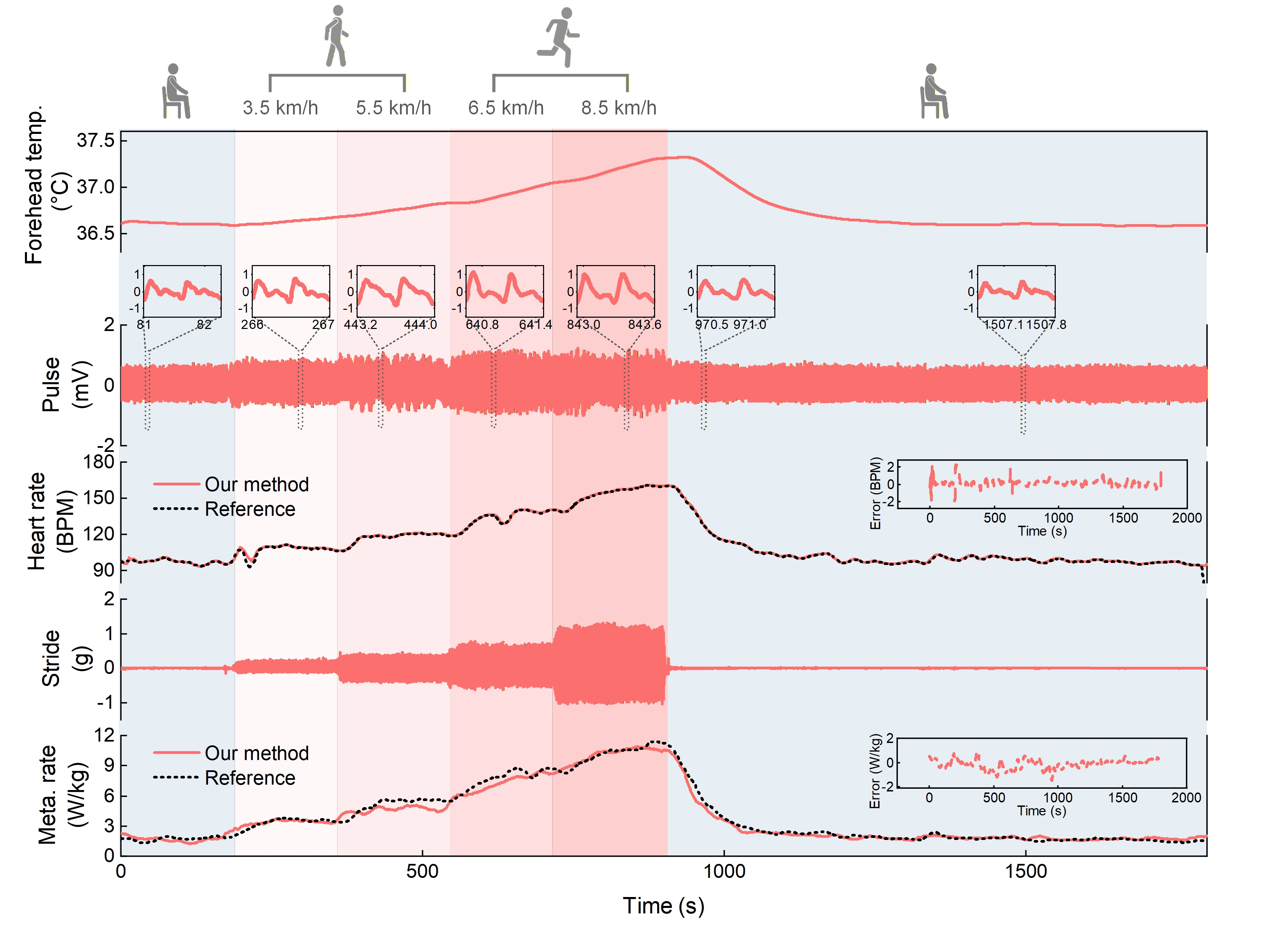
**

**Fig. S17. Results of multimodal physiological monitoring (Sub. 5).**

The insets show the detailed pulse waveforms of resting before exercise, slow walking, fast walking, slow running, fast running, stopping time of running, and resting after exercise. Meta. rate refers to the total metabolic energy cost of the paticipant per kilogram of body weight and per hour.

**
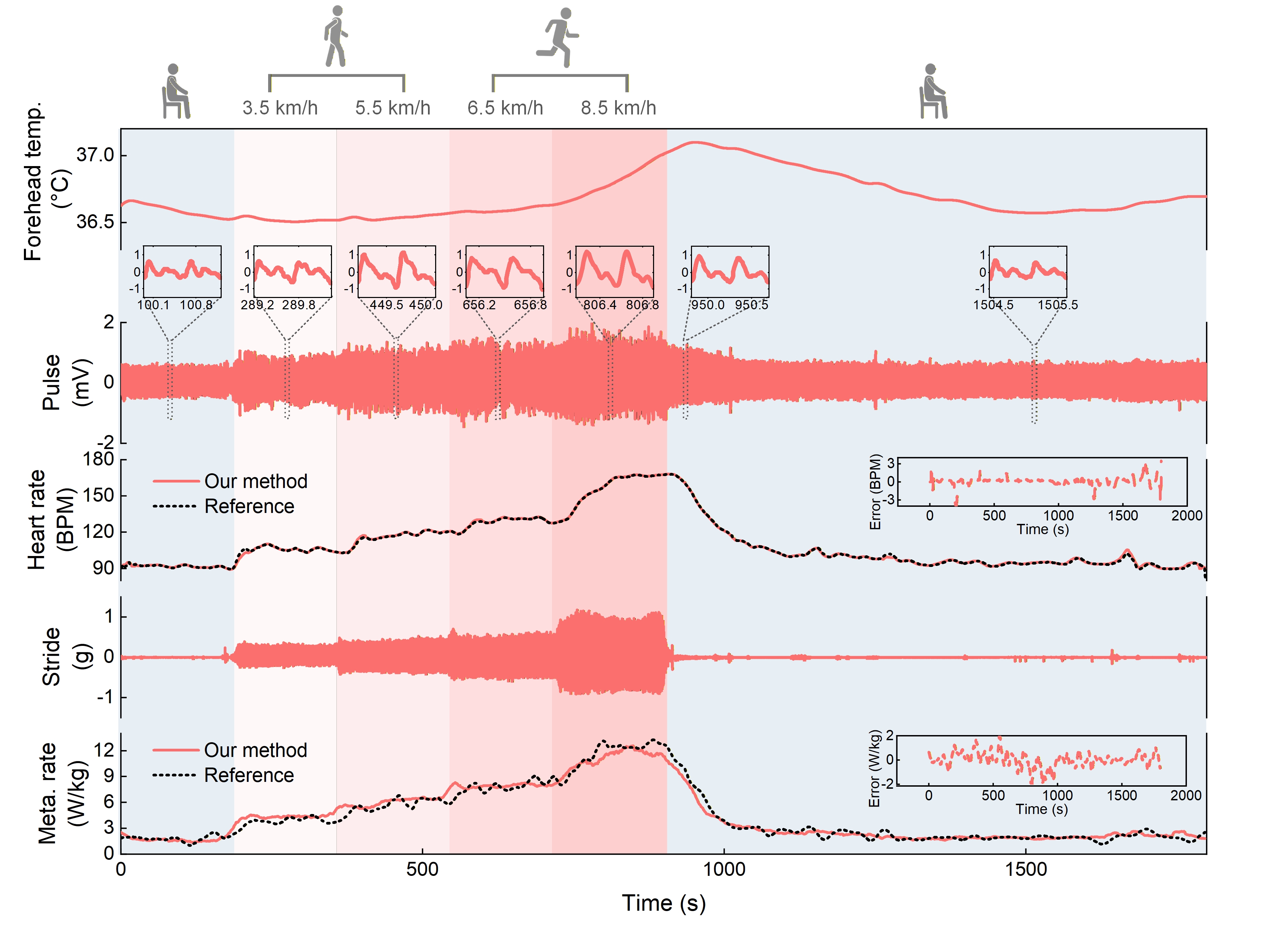
**

**Fig. S18. Results of multimodal physiological monitoring (Sub. 6).**

The insets show the detailed pulse waveforms of resting before exercise, slow walking, fast walking, slow running, fast running, stopping time of running, and resting after exercise. Meta. rate refers to the total metabolic energy cost of the paticipant per kilogram of body weight and per hour.

**
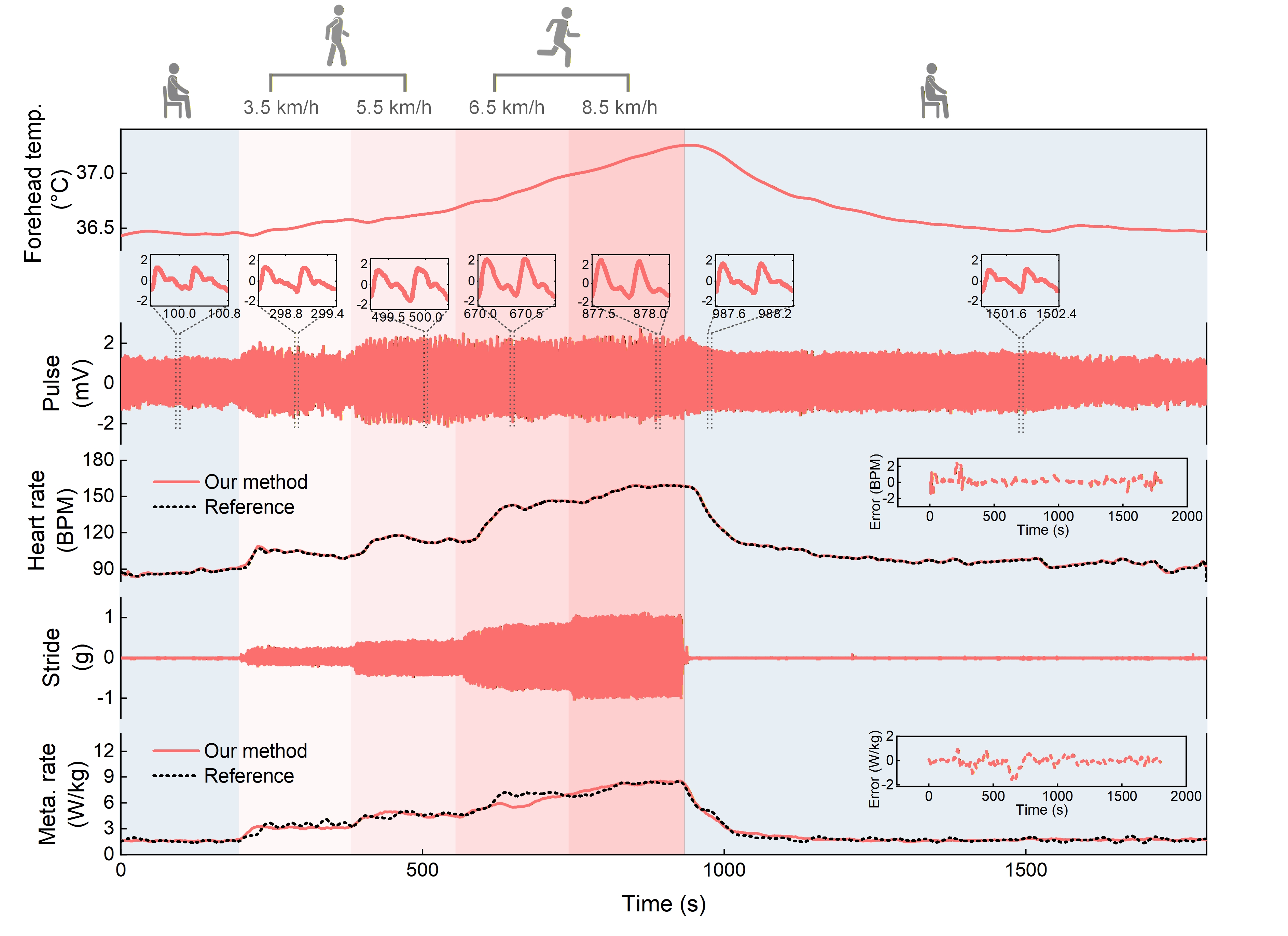
**

**Fig. S19. Results of multimodal physiological monitoring (Sub. 7).**

The insets show the detailed pulse waveforms of resting before exercise, slow walking, fast walking, slow running, fast running, stopping time of running, and resting after exercise. Meta. rate refers to the total metabolic energy cost of the paticipant per kilogram of body weight and per hour.

**
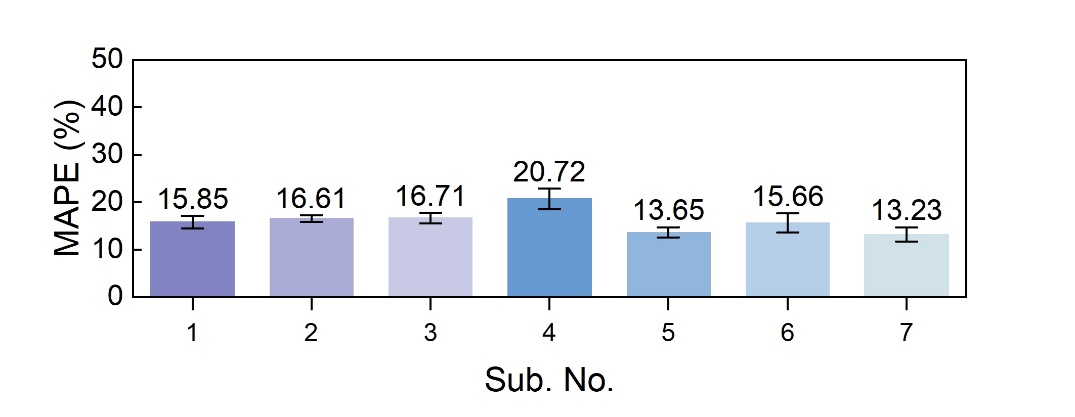
**

**Fig. S20. Generalizability validation of total metabolic rate estimation across 7 subjects.**

Datasets from 6 subjects are used for model training, and the remained subject is used for test. The average result of four independent trials of each tested subject is used for analysis. The error bar refers to the standard deviation.


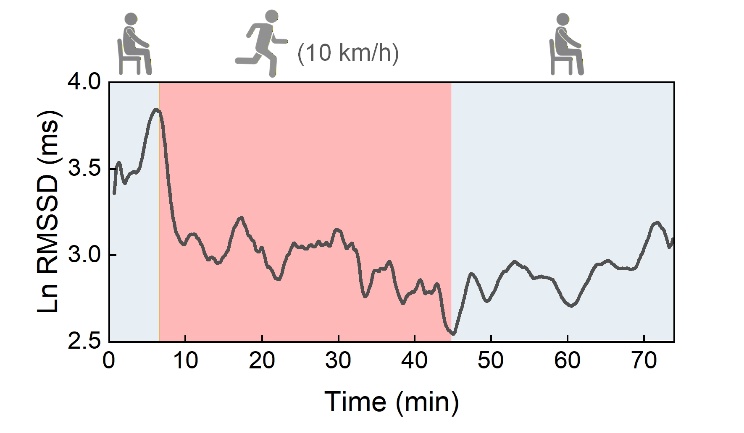


**Fig. S21. Fatigue level assessment by heart rate variability (HRV) during the long-term running.**

The natural logarithm of the root mean square of successive differences (Ln RMSSD) is used as the HRV metric. A lower Ln RMSSD indicates more suppressed parasympathetic nervous activity and a higher level of fatigue.


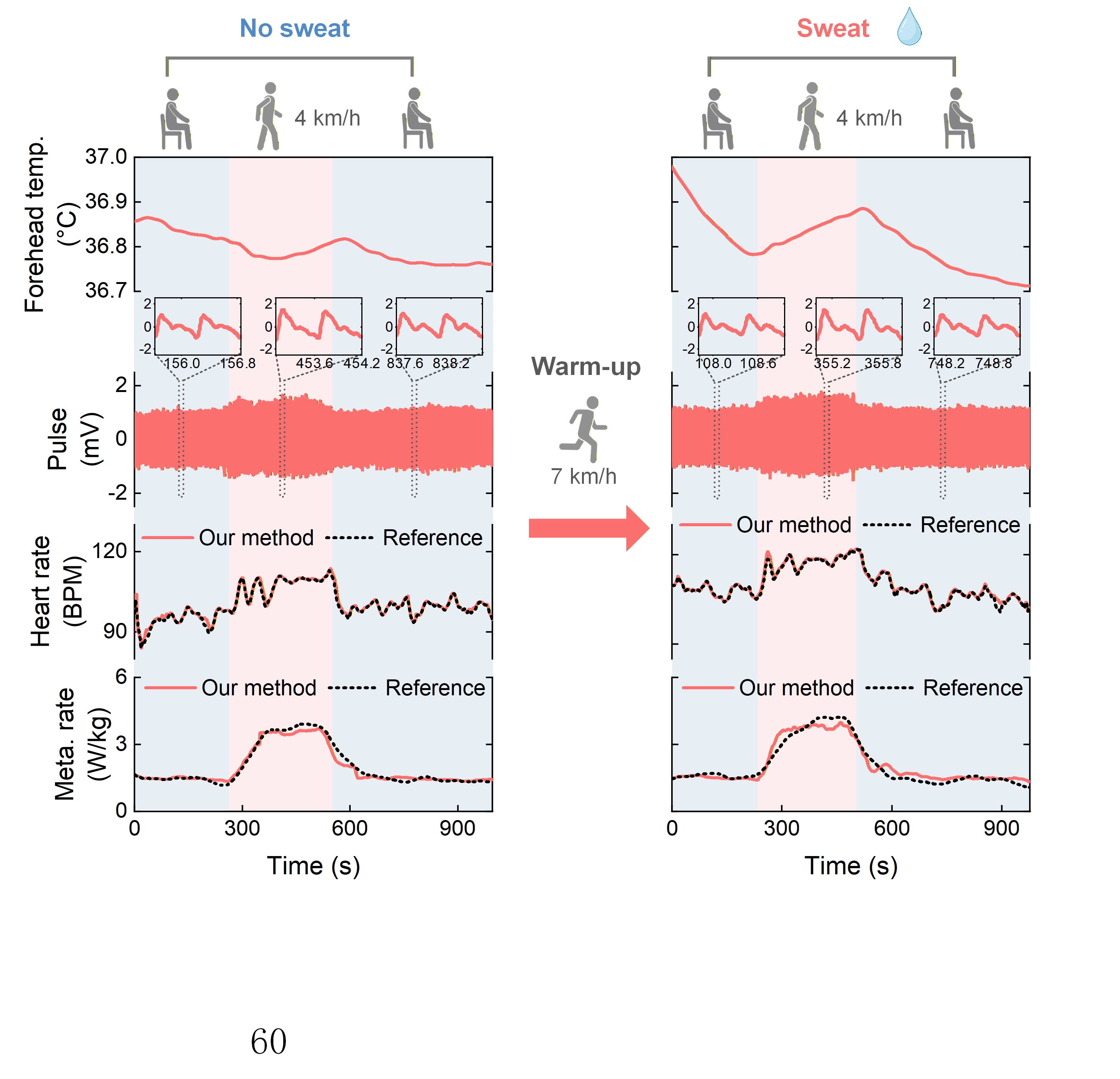


**Fig. S22. Results of multimodal physiological monitoring under sweating and non-sweating conditions.**

The insets show the detailed pulse waveforms during resting before exercise, walking, and resting after exercise. Meta. rate refers to the total metabolic energy cost of the paticipant per kilogram of body weight and per hour.


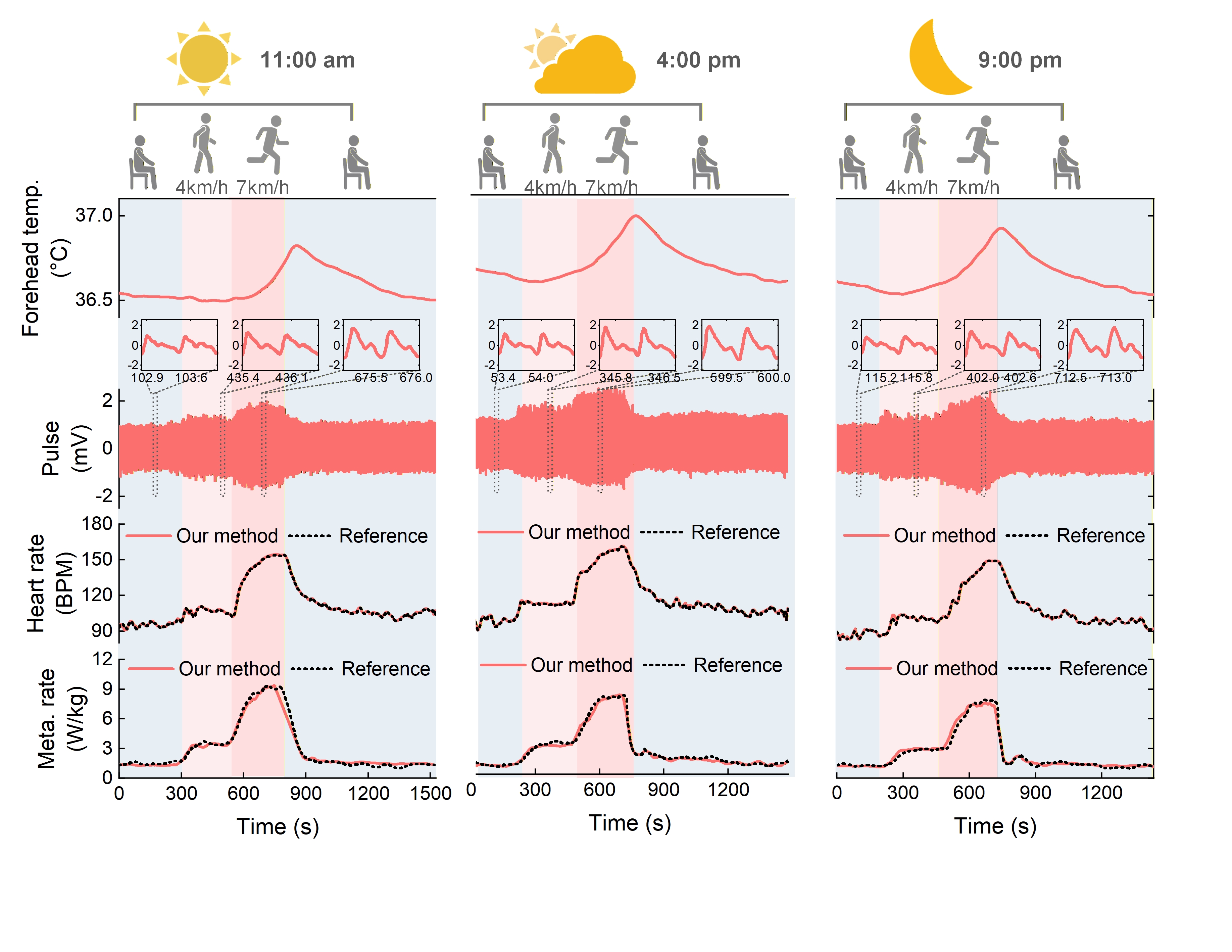


**Fig. S23. Three repeated measurements of multimodal physiological information within 1 day.**

The insets show the detailed pulse waveforms during resting before exercise, walking, and running. Meta. rate refers to the total metabolic energy cost of the paticipant per kilogram of body weight and per hour.

| **Sub. No.** | 1 | 2 | 3 | 4 | 5 | 6 | 7 | Mean | STD |
| --- | --- | --- | --- | --- | --- | --- | --- | --- | --- |
| AAE  (BPM) | 0.387 | 0.645 | 0.256 | 0.709 | 0.315 | 0.434 | 0.304 | 0.436 | 0.163 |

Table S1. The average absolute error of heart rate measurement for 7 subjects.

| **Sub. No.** | 1 | 2 | 3 | Mean | STD |
| --- | --- | --- | --- | --- | --- |
| ASE(℃) | 0.130 | 0.081 | 0.132 | 0.115 | 0.023 |
| Pearson Correlation | 0.974 | 0.896 | 0.916 | 0.929 | 0.033 |

Table S2. The average signed error and Pearson correlation coefficient of temperature measurement for 3 subjects.

| **No** | **Layer type** | **No. of filters** | **Kernel/**  **Pool size** | **Stride** | **Input size** | **Output size** | **Padding** |
| --- | --- | --- | --- | --- | --- | --- | --- |
| 1 | imageInput |  |  |  | (none, 101,9,3) | (none, 101,9,3) |  |
| 2 | convolution2d | 20 | (6,6) | (1,1) | (none, 101,9,3) | (none, 101,9,20) | same |
| 3 | relu |  |  |  | (none, 101,9,20) | (none, 101,9,20) |  |
| 4 | maxPooling2d |  | (4,4) | (1,1) | (none, 101,9,20) | (none, 101,9,20) | same |
| 5 | convolution2d | 30 | (3,3) | (1,1) | (none, 101,9,20) | (none, 101,9,30) | same |
| 6 | relu |  |  |  | (none, 101,9,30) | (none, 101,9,30) |  |
| 7 | maxPooling2d |  | (4,4) | (1,1) | (none, 101,9,30) | (none, 101,9,30) | same |
| 8 | flatten |  |  |  | (none, 101,9,30) | (none, 27270) |  |
| 9 | dropout |  |  |  | (none, 27270) | (none, 27270) |  |
| 10 | fullyConnected |  |  |  | (none, 27270) | (none, 1) |  |
| 11 | regression |  |  |  | (none, 1) | (none, 1) |  |

Table S3. The structure and hyperparameters of the CNN-based deep model for total metabolic rate estimation.

| **Sub. No.** | 1 | 2 | 3 | 4 | 5 | 6 | 7 | Mean | STD |
| --- | --- | --- | --- | --- | --- | --- | --- | --- | --- |
| MAPE  (%) | 8.12 | 10.38 | 11.28 | 13.71 | 10.13 | 7.50 | 9.53 | 10.09 | 1.91 |

Table S4. Individual validation of total metabolic rate estimation for 7 subjects.

Seven subjects were aged between 21 and 35 years, including six males and one female. Four independent trials are conducted, of which 3 repetitions are for model training and the remained repetition is for model test. Leave-One-Out cross validation is used, and the average performance is used for analysis. MAPE refers to the average mean absolute percentage error.

| **Sub. No.** | 1 | 2 | 3 | 4 | 5 | 6 | 7 | Mean | STD |
| --- | --- | --- | --- | --- | --- | --- | --- | --- | --- |
| MAPE  (%) | 15.85 | 16.61 | 16.71 | 20.72 | 13.65 | 15.66 | 13.23 | 16.06 | 1.41 |

**Table S5. Generalizability validation of total metabolic rate estimation across 7 subjects.**

Seven subjects were aged between 21 and 35 years, including six males and one female. Datasets from 6 subjects are used for model training, and the remained subject is used for test. The average result of four independent trials of each tested subject is used for analysis. MAPE refers to the average mean absolute percentage error.

| **Sensing principle** | **Steady pulse** | **Exercise pulse** | **Steady heart rate** | **Exercise heart rate**  **(AAE)** | **Steady body temperature**  **（ASE / r）** | **Exercise body temperature**  **（ASE / r）** | **Static metabolism**  **（MAPE /**$\mathbf{r}^{\mathbf{2}}$**）** | **Exercise metabolism**  **（MAPE /**$\mathbf{r}^{\mathbf{2}}$**）** | **Response time (s)** | **Ref** |
| --- | --- | --- | --- | --- | --- | --- | --- | --- | --- | --- |
| NTC thermistors / IMU | \ | \ | Yes | \ | -0.05 / 1 | \ | \ | \ | 7.6 | 17 |
| Thermal flux | \ | \ | \ | \ | N.A. / 0.80 | N.A. / 0.80 | \ | \ | 25 | 19 |
| Thermal flux | \ | \ | \ | \ | \ | 0.23 / N.A. | \ | \ | 1.6 | 20 |
| Temperature / Humidity | \ | \ | Yes | \ | \ | \ | N.A. / 0.9 | N.A. / 0.9 | \ | 14 |
| IMU | \ | \ | \ | \ | \ | \ | \ | 13% / N.A. | \ | 24 |
| Optical PPG | Yes | \ | Yes | 1.75 | \ | \ | \ | \ | 0.056 | 32 |
| ECG | \ | \ | Yes | \ | \ | \ | \ | \ | \ | 48 |
| Pressure | Yes | \ | Yes | \ | \ | \ | \ | \ | \ | 49 |
| Ultrasonic | Yes | \ | Yes | \ | \ | \ | \ | \ | \ | 50 |
| **Thermal conductivity** | **Yes** | **Yes** | **Yes** | **0.44** | **0.11 / 0.98** | **0.11 / 0.93** | **12.67% / 0.98** | **7.67% / 0.98** | **0.06** | **This**  **work** |

**Table S6. The comparison of multimodal physiological monitoring between our MetaBand and other existing wearable methods.**

| **State** | No sweat | Sweat | Mean | STD |
| --- | --- | --- | --- | --- |
| **Heart rate AAE**  **（BPM）** | 0.521 | 0.468 | 0.495 | 0.027 |
| **Meta. rate MAPE**  **(%)** | 11.85 | 12.04 | 11.95 | 0.10 |

**Table S7. The AAE of heart rate measurement and MAPE of total metabolic rate estimation under sweating and non-sweating conditions.**

| **Time** | 11:00 am | 4:00 pm | 9:00 pm | Mean | STD |
| --- | --- | --- | --- | --- | --- |
| **Heart rate AAE**  **（BPM）** | 0.393 | 0.616 | 0.511 | 0.507 | 0.091 |
| **Meta. rate MAPE**  **(%)** | 12.43 | 12.15 | 12.66 | 12.41 | 0.21 |

**Table S8. The AAE of heart rate measurement and MAPE of total metabolic rate estimation during the 1-day experiments.**

Movie S1. Demonstration of the Metaband for real-time multimodal physiological monitoring during indoor exercise. HR refers to heart rate, TEE refers to total energy expenditure, and SF refers to stride frequency.

Movie S2. Demonstration of the Metaband for real-time multimodal physiological monitoring during outdoor activities. HR refers to heart rate, TEE refers to total energy expenditure, and SF refers to stride frequency.
